# *Flavonifractor Plautii* or Its Metabolite Desaminotyrosine as Prophylactic Agents for Alleviating Myocardial Ischemia/Reperfusion Injury

**DOI:** 10.1002/advs.202417827

**Published:** 2025-03-16

**Authors:** Heng Du, Xu Liu, Jianghua Shen, Hailong Yuan, Hao Zhang, Gan Xi, Yujing Li, Yuhan Wang, Jiahe Zhang, Chaofan Yang, Pengfei Xu, Jiawan Wang, Fang Wang, Siqi Liu, Yanan Zhou, Qi Gu, Jingjing Lu, Tuo Wei, Zeyu Gao, Jingyi Zang, Jun Wang, Moshi Song

**Affiliations:** ^1^ State Key Laboratory of Organ Regeneration and Reconstruction Institute of Zoology Chinese Academy of Sciences Beijing 100101 China; ^2^ University of Chinese Academy of Sciences Beijing 100049 China; ^3^ CAS Key Laboratory of Pathogenic Microbiology and Immunology Chinese Academy of Sciences Beijing 100101 China; ^4^ Beijing Institute for Stem Cell and Regenerative Medicine Beijing 100101 China; ^5^ Institute for Stem Cell and Regeneration Chinese Academy of Sciences Beijing 100101 China; ^6^ Department of Anesthesiology Beijing Chao‐Yang Hospital Beijing 100020 China

**Keywords:** cardiac inflammation, cardiomyocyte survival, desaminotyrosine, *Flavonifractor plautii*, myocardial ischemia/reperfusion injury

## Abstract

Myocardial ischemia/reperfusion (I/R) injury is a major contributor to myocardial damage, leading to adverse cardiac remodeling and dysfunction. Recent studies have highlighted the potential of gut microbiota‐derived metabolites in modulating cardiac outcomes. Here, the cardioprotective effects of a commensal bacterium *Flavonifractor plautii* (*F. plautii*) and its metabolite desaminotyrosine (DAT) against myocardial I/R injury are investigated. We showed that prophylactic gavage of *F. plautii* attenuates myocardial I/R injury as evidenced by improved cardiac function and reduced cardiac injury. We also found that its metabolite DAT recapitulates these cardioprotective effects against myocardial I/R injury. Transcriptomic analysis has revealed that DAT preserves cardiac tissue and attenuates immune responses against myocardial I/R injury. Mechanistically, DAT promotes cardiomyocyte survival through the modulation of the nicotinamide adenine dinucleotide phosphate (NADP^+^/NADPH) ratio. Further, DAT suppressed macrophage proinflammatory activities and cardiac inflammation via the reduction in interleukin‐6 (IL‐6) production. Taken together, our findings indicate that *F. plautii* and its metabolite DAT exert pleiotropic cardioprotective effects against myocardial I/R injury, suggesting them as potential prophylactic therapeutic options for alleviating myocardial I/R injury.

## Graphical abstract

The gut microbiome is a potential target for treating cardiovascular disease. It is found that prophylactic administration of *Flavonifractor plautii* or its metabolite desaminotyrosine (DAT) exerts pleiotropic cardioprotective effects via promoting cardiomyocyte survival and attenuating cardiac inflammation against myocardial ischemia/reperfusion (I/R) injury. The findings suggest that *Flavonifractor plautii* and DAT may serve as prophylactic strategies for alleviating myocardial I/R injury.

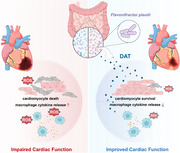

## 1 Introduction

Cardiovascular disease (CVD), including ischemic heart disease (IHD) and stroke, remains the predominant cause of global morbidity and mortality, with myocardial infarction (MI) being a particularly severe and life‐threatening manifestation.^[^ ^1^, ^2^ ^]^ As a common sequence of MI following percutaneous coronary intervention (PCI) and thrombolytic therapy, myocardial ischemia/reperfusion (I/R) injury aggravates the initial myocardial damage and is estimated to contribute to approximately 50% of the final infarct size.^[^ ^3^, ^4^ ^]^ Recognizing the complexity and severe outcomes associated with I/R injury, there is an urgent need to develop strategies that can effectively reduce the detrimental effects of I/R injury.

Given the substantial evidence highlighting the gut microbiome's impact on cardiovascular health, there is a compelling rationale to investigate the modulation of the microbiome or the use of specific bacteria as a therapeutic approach for myocardial I/R injury.^[^ ^5^, ^6^, ^7^ ^]^ The gut microbiome's role in cardiovascular diseases is multifaceted,^[^ ^8^ ^]^ with its metabolites, such as trimethylamine N‐oxide (TMAO)^[^ ^9^, ^10^ ^]^ and short‐chain fatty acids (SCFA),^[^ ^11^, ^12^ ^]^ being directly implicated in the pathogenesis of conditions like hypertension,^[^ ^9^, ^13^ ^]^ heart failure,^[^ ^14^, ^15^ ^]^ and MI.^[^ ^16^, ^17^ ^]^ Studies have underscored the promise of probiotics in mitigating I/R injury, particularly strains such as *Lactobacillus reuteri* with its metabolite gamma‐aminobutyric acid (GABA)^[^ ^6^ ^]^ and *Bifidobacterium infantis* with its metabolite inosine^[^ ^5^ ^]^ that we have previously reported. Yet, given the complexity of myocardial I/R injury and its diverse pathological characteristics, it is crucial to identify a wider array of bacteria that can mitigate I/R injury, to uncover more diverse and potent therapeutic options, and to meet the unique needs of individual patients given the high variability in gut microbiome composition among different individuals.^[^ ^18^ ^]^ *Flavonifractor plautii* (*F. plautii*) has been recognized for its anti‐inflammatory effects, which help prevent inflammation and are linked to a lower risk of intestinal and metabolic disorders.^[^ ^19^, ^20^, ^21^ ^]^ A recent study indicates that *F. plautii* plays a role in protecting against elevated arterial stiffness by inhibiting metalloproteinase activity,^[^ ^22^ ^]^ further highlighting its importance in human health beyond the gut. However, it is unclear whether *F. plautii* could be used to mitigate I/R injury.

In this study, we showed that prophylactic *F. plautii* gavage attenuated myocardial I/R injury, which was recapitulated by its metabolite desaminotyrosine (DAT). Mechanistically, DAT enhanced cardiomyocyte survival via antioxidant actions via the modulation of the nicotinamide adenine dinucleotide phosphate (NADP^+^/NADPH) ratio. Additionally, DAT suppressed the proinflammatory activities of macrophage by suppressing interleukin‐6 (IL‐6) production and therefore attenuated I/R‐induced cardiac inflammation. Our study demonstrates that prophylactic *F. plautii* gavage confers cardioprotection against I/R injury, in particular through its metabolite DAT which has a combinatory effect of enhancing cardiomyocyte survival and suppressing cardiac inflammation, thus proposing their potentials as therapeutic strategies for the management of myocardial I/R injury.

## 2 Results

### 2.1 Prophylactic Gavage of *F. plautii* Attenuated Myocardial I/R Injury

**Figure 1:**
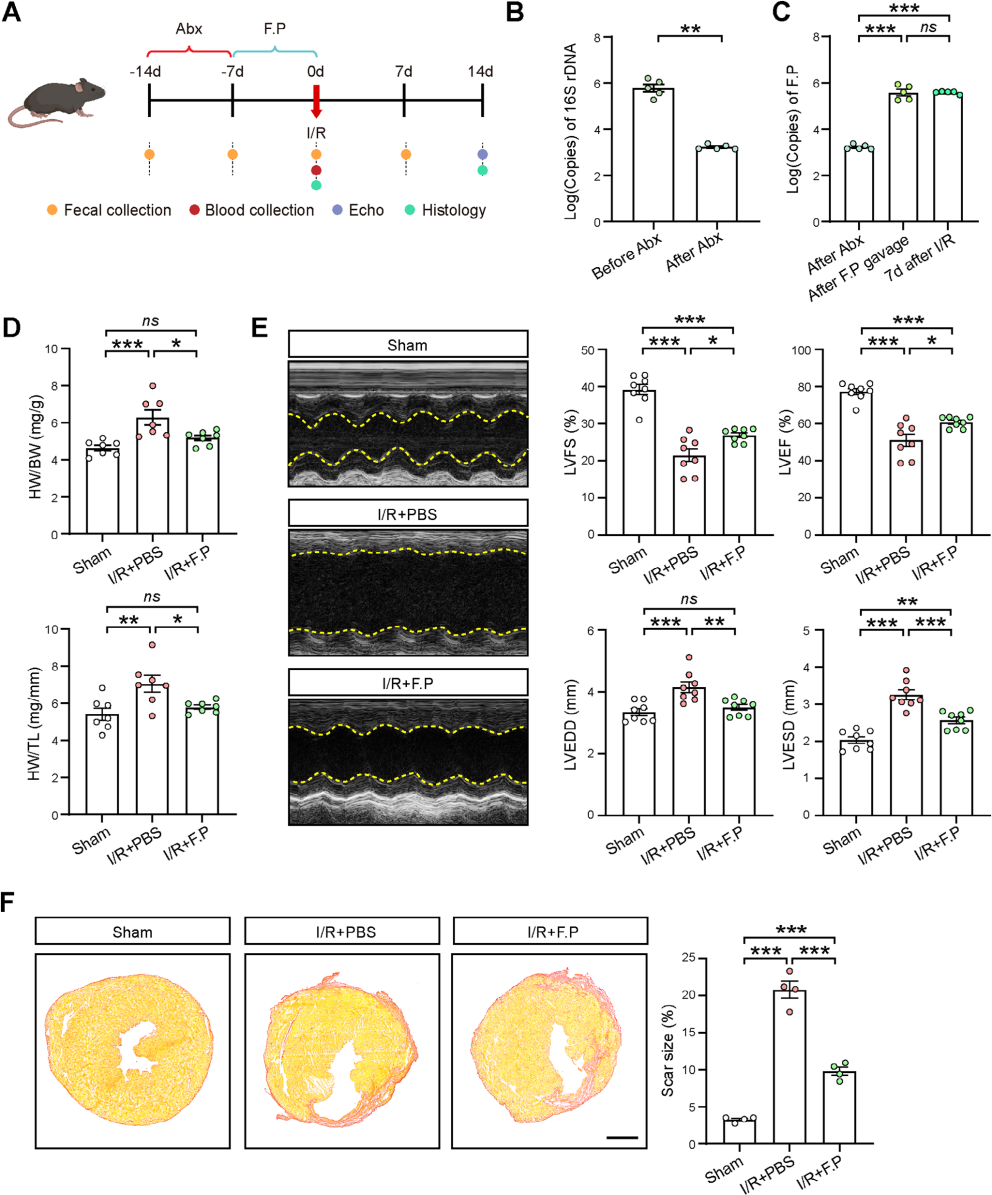
Prophylactic *F. plautii* gavage attenuated cardiac dysfunction in mouse hearts after I/R. A) Schematic diagram of the experiment with *F. plautii* (F.P) gavage. B) The copies of bacterial 16S rDNA detected in the feces collected before and after antibiotics (Abx) treatment. *n = *5 for each group. C) The copies of *F. plautii* rDNA detected in the feces collected after antibiotics (Abx) treatment, after 1 week *F. plautii* gavage, and 7 d after I/R. *n = *5 for each group. D) The ratios of heart weight to body weight (HW/BW) and heart weight to tibial length (HW/TL) in the Sham, PBS‐gavaged I/R (I/R+PBS), and *F. plautii*‐gavaged I/R (I/R+F.P) groups at 2 weeks after the surgery. *n = *7 for each group. E) Representative echocardiographic images at 2 weeks after I/R (left panel). Quantitative data of left ventricular fractional shortening (LVFS), left ventricular ejection fraction (LVEF), left ventricular end‐diastolic dimension (LVEDD), and left ventricular end‐systolic dimension (LVESD) at 2 weeks after I/R (right panels). *n = *8 for each group. F) Picrosirius red staining of transverse cardiac sections from hearts at 2 weeks after I/R. Scale bar, 1 mm. Quantifications of fibrotic scar tissues are shown in the right panel. *n = *4 for each group. Quantitative data are presented as mean ± SEM. Groups were compared using B) Student's *t*‐test or C–F) one‐way ANOVA. *ns*, not significant; *p < 0.05; **p < 0.01; ***p < 0.001.

Previous research has indicated that supplementing with *F. plautii* can mitigate atherosclerosis by suppressing matrix metalloproteinase activity, preventing the rupture of elastic fibers, and curbing localized vascular inflammation, thereby exerting anti‐inflammatory effects.^[^ ^22^ ^]^ Recognizing the significance of anti‐inflammatory properties in the management of cardiovascular diseases,^[^ ^23^ ^]^ here we investigated the potential cardioprotective effects by prophylactic gavage of *F. plautii* in protecting against myocardial I/R injury (**Figure** **1**A). Following the depletion of the gut microbiota using orally administered antibiotics for 7 d (Figure 1B), 8‐week‐old male C57BL/6J mice were subjected to a daily gavage of *F. plautii* (5 × 10^8^ colony‐forming units (CFU) d^−1^) or phosphate buffered saline (PBS) (control group) for additional 7 d. The colonization of the *F. plautii* was validated by quantifying 16S ribosomal DNA (16S rDNA) (Figure 1C). Subsequently, myocardial I/R injury was induced by temporarily occluding the left anterior descending (LAD) coronary artery for 40 min followed by timely reperfusion, with a Sham group subjected to a similar operational procedure excepting LAD occlusion serving as the control for this procedure.

A significant hypertrophic response was noted in the PBS‐gavaged I/R mice compared to the Sham group at 2 weeks after the surgery, with a significant increase in both the heart weight to body weight (HW/BW) ratio and the heart weight to tibial length (HW/TL) ratio, which was significantly mitigated in the *F. plautii*‐gavaged I/R mice (Figure 1D). We subsequently assessed the potential cardioprotective effects by prophylactic gavage of *F. plautii* against I/R‐induced injury. Thoracic echocardiography was employed to evaluate cardiac contractility and left ventricular (LV) chamber dimensions at 2 weeks after the surgery. A significant reduction in cardiac contractility and an enlargement of the LV chamber were observed in the PBS‐gavaged I/R group when compared to the Sham group. In contrast, *F. plautii*‐gavaged I/R group exhibited significantly improved left ventricular fractional shortening (LVFS%) and ejection fraction (LVEF%), along with reduced left ventricular end‐diastolic dimension (LVEDD) and left ventricular end‐systolic dimension (LVESD) compared to the PBS‐gavaged I/R group (Figure 1E). Additionally, we performed a histopathological analysis of cardiac tissues across all groups. Picrosirius red staining indicated that, compared to the Sham group, hearts from PBS‐gavaged I/R mice had significantly larger fibrotic scar tissues, while these fibrotic scar tissues were significantly smaller in the *F. plautii*‐gavaged I/R group (Figure 1F). Taken together, these findings suggest that prophylactic gavage of *F. plautii* confers cardioprotection against I/R‐induced cardiac injury.

### 2.2 DAT Recapitulated the Cardioprotective Effect of *F. plautii* against Myocardial I/R Injury

**Figure 2:**
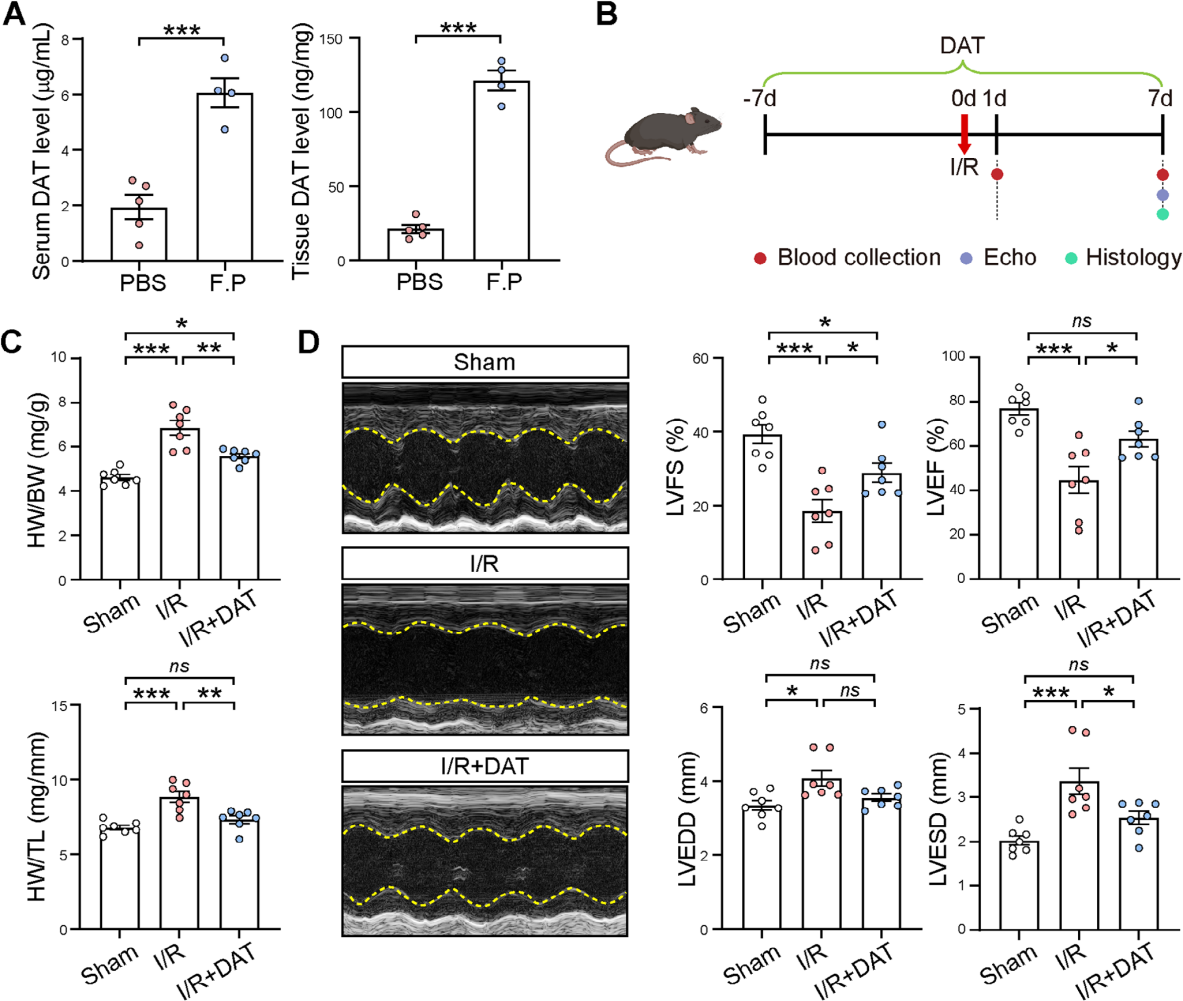
DAT recapitulated the cardioprotective effects of *F. plautii* in mouse hearts after I/R. A) Levels of desaminotyrosine (DAT) measured in the serum (left panel) and heart tissues (right panel) by HPLC‐MS after 1 week PBS and *F. plautii* (F.P) gavage. *n = *5 for PBS group; *n = *4 for F.P group. B) Schematic diagram of mouse experiment with DAT treatment at a final concentration of 20 mg mL^−1^ in drinking water and I/R surgery. C) The ratios of HW/BW and HW/TL in the Sham, I/R, and DAT‐treated I/R (I/R+DAT) groups at 1 week after the surgery. *n = *7 for each group. D) Representative echocardiographic images at 1 week after I/R are shown in the left panel. *n = *7 for each group. Quantitative results of LVFS, LVEF, LVEDD, and LVESD are shown in the right panel. Data are presented as mean ± SEM. Groups were compared using A) Student's *t*‐test or C,D) one‐way ANOVA. *ns*, not significant; *p < 0.05; **p < 0.01; ***p < 0.001.

As a commensal bacterium in the human gut, *F. plautii* is capable of breaking down dietary flavonoids into monophenolic acids including DAT (Figure S1, Supporting Information), which possess antioxidant and anti‐inflammatory activities.^[^ ^24^, ^25^, ^26^, ^27^ ^]^ We hypothesized that DAT may contribute to the cardioprotective effects observed with the prophylactic gavage of *F. plautii*. Accordingly, we measured the DAT levels in both the PBS‐gavaged I/R and *F. plautii*‐gavaged I/R groups and observed approximately 3.5‐fold increase in serum DAT levels in *F. plautii*‐gavaged mice compared to the PBS‐gavaged group (**Figure** **2**A). Similarly, DAT levels in heart tissues were significantly elevated in *F. plautii*‐gavaged mice compared to the PBS‐gavaged group (Figure 2A).

We then investigated the potential cardioprotective effects of DAT in I/R mice against myocardial I/R injury (Figure 2B). DAT administered via drinking water at a final concentration of 20 mg mL^−1^ resulted in approximately 3.4‐fold increase in serum DAT levels compared to controls without DAT treatment, which was comparable to the aforementioned increase achieved through gavage with *F. plautii*. Our results showed that DAT administration mirrored the positive outcomes observed in *F. plautii*‐gavaged mice. Notably, DAT significantly reduced I/R‐induced cardiac hypertrophy, as indicated by the increased HW/BW and HW/TL ratios post‐I/R, which were reversed by DAT treatment (Figure 2C). Further, DAT alleviated cardiac dysfunction induced by I/R, demonstrated by significantly improved LVFS, LVEF, and reduced LVEDD and LVESD at 1 week after the surgery (Figure 2D). Collectively, our data indicate that DAT reproduces the cardioprotective effects of *F. plautii* against I/R‐induced cardiac injury.

### 2.3 DAT Preserved Cardiac Tissue and Attenuated Immune Response in I/R‐Injured Hearts

**Figure 3:**
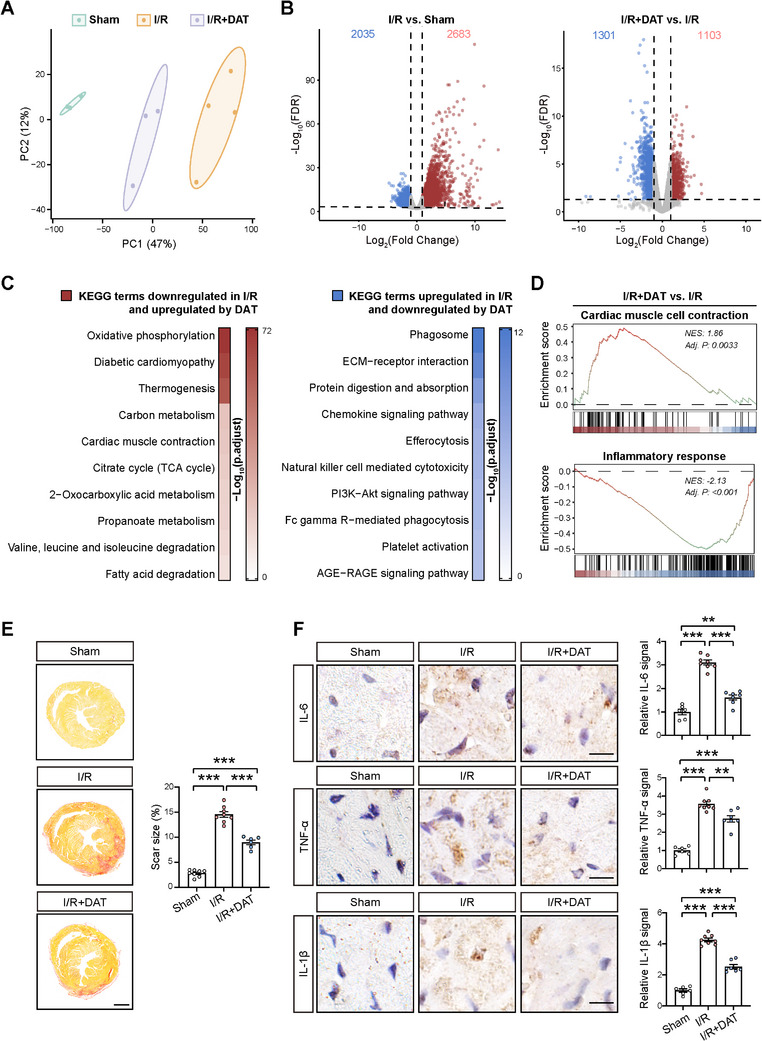
DAT preserved cardiac tissue and attenuated immune response in mouse hearts after I/R. A) Principal component analysis (PCA) on the transcriptome data obtained from injured heart tissues of mice in the I/R and DAT‐treated I/R (I/R+DAT) groups at 1 week after the surgery, along with the transcriptome data obtained from corresponding tissue samples from the Sham group at 1 week after the surgery. B) Volcano plot of genes differentially regulated in I/R versus Sham groups (left panel) and I/R+DAT versus I/R groups (right panel). The blue dots denote downregulated genes, the red dots denote upregulated genes, and the grey dots denote genes without significantly differential expression. C) KEGG analysis of pathways associated with genes downregulated in I/R versus Sham groups and upregulated in I/R+DAT versus I/R groups (indicated in red, left panel) and pathways associated with genes upregulated in I/R versus Sham groups and downregulated in I/R+DAT versus I/R groups (indicated in blue, right panel). D) GSEA plots showing the enrichment of gene sets related to cardiac muscle cell contraction (upper panel) and inflammatory response (lower panel) in I/R+DAT versus I/R groups. E) Picrosirius red staining of transverse cardiac sections from hearts at 1 week after I/R. Scale bar, 1 mm. Quantifications of fibrotic scar tissues are shown in the right panel. *n = *8 for Sham and I/R groups; *n = *6 for I/R+DAT group. F) Immunohistochemical staining for IL‐6 (upper panel), TNF‐α (middle panel), and IL‐1β (lower panel) at 1 week after I/R. Scale bar, 10 µm. Quantitative data are shown to the right. *n = *6 for Sham group; *n = *8 for I/R group; *n = *7 for I/R+DAT group. Data are presented as mean ± SEM. E,F) Groups were compared using one‐way ANOVA. **p < 0.01; ***p < 0.001.

To elucidate the mechanisms by which DAT treatment confers cardioprotection against myocardial I/R injury, we conducted a comparative transcriptomic analysis of infarcted heart tissues from mice in the Sham group, I/R group, and DAT‐treated I/R group at 1 week after the surgery (Figure **3**A). RNA sequencing analysis identified 2035 downregulated genes and 2683 upregulated in the I/R group relative to the Sham group (Figure 3B). Notably, DAT treatment reversed the expression of 1021 I/R‐downregulated genes and of 1272 I/R‐upregulated genes (Figure S2A, Supporting Information). Subsequently, Kyoto Encyclopedia of Genes and Genomes (KEGG) analysis revealed that among the top DAT‐upregulated pathway, several were aligned with the preservation of cardiac tissue, such as cardiac muscle contraction and fatty acid degradation, suggesting that DAT helps maintain cardiac muscle function under I/R conditions (Figure 3C). Conversely, among the top DAT‐downregulated pathways, several were associated with inflammatory responses, including the Fc gamma receptor‐mediated phagocytosis and chemokine signaling pathways, pointing to an anti‐inflammatory cardioprotective effect of DAT (Figure 3C). Consistent with these findings, gene set enrichment analysis (GSEA) data indicated that cardiac muscle cell contraction was downregulated after I/R but was rescued by DAT treatment. Similarly, the inflammatory response, which was upregulated after I/R, was mitigated by DAT treatment (Figure 3D and Figure S2B, Supporting Information). Therefore, our transcriptional data suggest that DAT confers cardioprotection by preserving cardiac tissue and attenuating immune response.

Consistent with the RNA sequencing data indicating preserved cardiac tissue by DAT treatment after I/R, picrosirius red staining revealed substantial scar tissues in hearts from I/R mice, which were significantly smaller in the DAT‐treated I/R group at 1 week after the surgery (Figure 3E). By contrast, align with the RNA sequencing data revealing an anti‐inflammatory effect of DAT treatment after I/R, we examined the expression of IL‐6,^[^ ^28^ ^]^ tumor necrosis factor alpha (TNF‐α), and interleukin‐1β (IL‐1β),^[^ ^29^, ^30^ ^]^ which are proinflammatory cytokines that are upregulated after I/R. Our immunohistochemical analysis revealed that the I/R‐induced increases in the expression of these proinflammatory cytokines were significantly reduced by DAT treatment (Figure 3F). Together, these data suggest that DAT attenuated immune response and preserved cardiac muscle in I/R‐injured hearts.

### 2.4 DAT Promoted Cardiomyocyte Survival by Modulating the NADP^+^/NADPH Ratio

**Figure 4:**
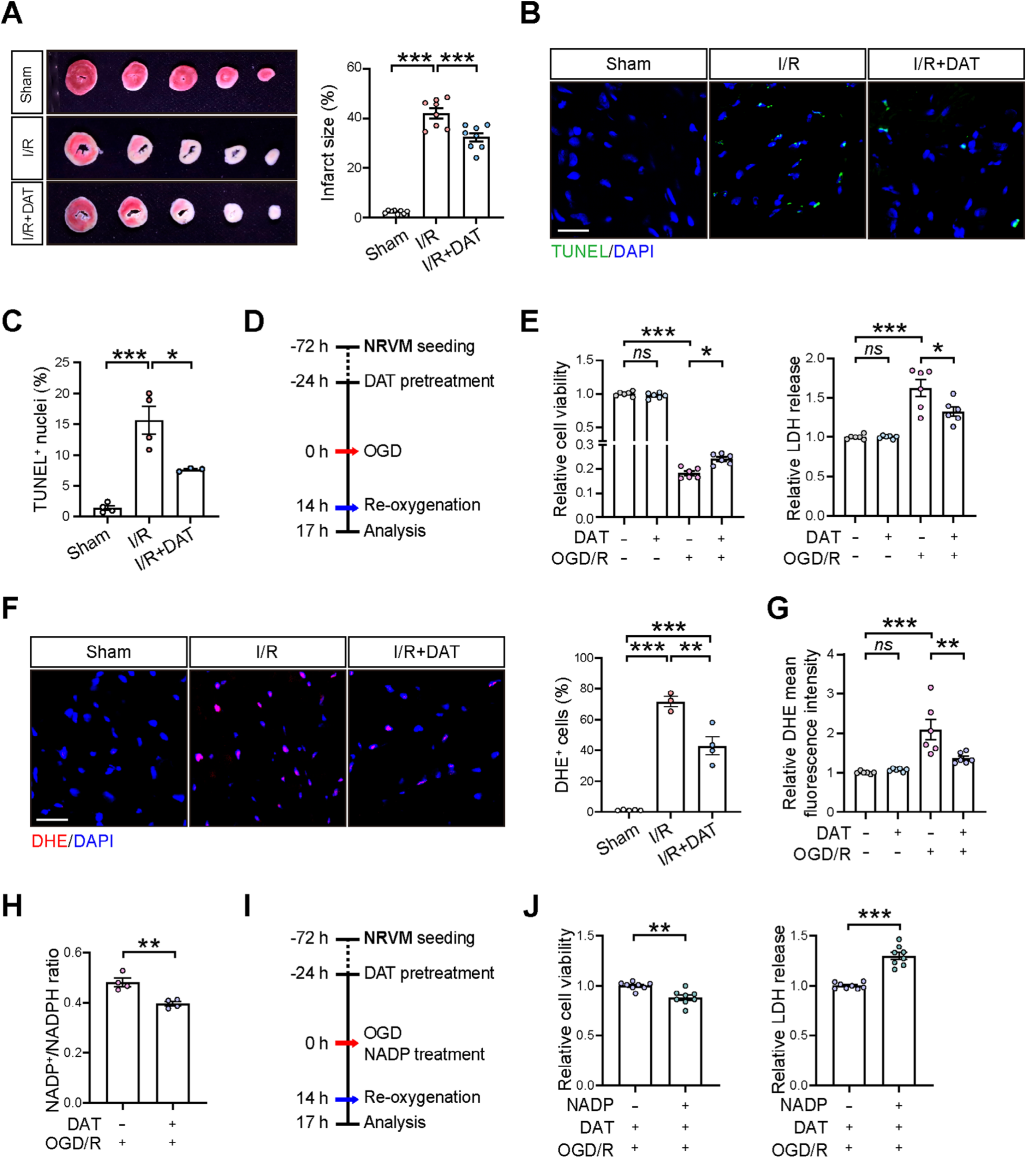
DAT promoted cardiomyocyte survival by modulating the NADP^+^/NADPH ratio. A) Triphenyltetrazolium chloride (TTC) staining of heart sections collected from Sham, I/R, and DAT‐treated I/R (I/R+DAT) groups at 1 d after the surgery. *n = *8 mice for each group. B) Representative images and C) quantification of TUNEL staining in heart sections from Sham, I/R, and I/R+DAT groups at 1 d after the surgery. TUNEL (green) and DAPI (blue). Scale bar, 20 µm. *n = *4 for Sham and I/R groups; *n = *3 for I/R+DAT group. D) Schematic diagram illustrating the in vitro experiments with neonatal rat ventricular cardiomyocytes (NRVMs) subjected to DAT pretreatment at 100 µm followed by oxygen glucose deprivation/reoxygenation (OGD/R) that simulated myocardial I/R injury. E) Cell viability measured by Alamar blue staining of NRVMs (left panel) and LDH (lactate dehydrogenase) release from NRVMs (right panel) in the presence or absence of DAT under normoxic and OGD/R conditions. *n = *6 for each group. F) DHE staining in heart sections from Sham, I/R, and I/R+DAT groups. Scale bar, 20 µm. *n = *5 for Sham group; *n = *3 for I/R group; *n = *4 for I/R+DAT group. DHE (red) and DAPI (blue). G) DHE staining of NRVMs in the presence or absence of DAT under normoxic and OGD/R conditions. *n = *6 for each group. H) The ratio of NADP^+^/NADPH calculated in the presence or absence of DAT under OGD/R condition. *n = *4 for each group. I) Schematic diagram illustrating the in vitro experiments with NRVM subjected to DAT pretreatment at 100 µm followed by OGD/R in the presence or absence of oxidized NADP^+^. J) Cell viability measured by Alamar blue staining of NRVMs (left panel) and LDH release from NRVMs (right panel). *n = *8 for each group. Quantitative data are presented as mean ± SEM. Groups were compared using A,C,F) one‐way ANOVA, E,F) two‐way ANOVA or H,J) Student′s *t*‐test. *ns*, not significant; *p < 0.05; **p < 0.01; ***p < 0.001.

In line with RNA sequencing data showing that DAT treatment maintained cardiac tissue integrity at 1 week after I/R, we observed that DAT treatment reduced I/R‐induced infarct size, as indicated by increased areas of unstained cardiac tissues by 2,3,5‐triphenyltetrazolium chloride (TTC) staining in I/R mice compared to the Sham group at 1 d after the surgery, which was reversed in DAT‐treated I/R mice (**Figure** **4**A). We also observed increased terminal deoxynucleotidyl transferase‐mediated dUTP nick end labeling (TUNEL) positivity in I/R mice compared to the Sham group at 1 d after the surgery, which was again significantly mitigated by DAT treatment (Figure 4B,C). Further, to elucidate the protective mechanisms of DAT by which preserved cardiac muscle following I/R, we utilized an in vitro model with neonatal rat ventricular myocytes (NRVMs), NIH3T3 fibroblasts, and mouse cardiac endothelial cells (MCECs), which were subjected to oxygen glucose deprivation/reoxygenation (OGD/R) to mimic in vivo I/R injury (Figure 4D and Figure S3, Supporting Information). We discovered that DAT's prosurvival effect was specific to NRVMs (Figure 4E); DAT did not enhance survival or primary functions in NIH3T3 fibroblasts and MCECs (Figure S3, Supporting Information). These findings suggest that the preservation of cardiac tissue by DAT treatment is primarily attributed to its prosurvival effect on cardiomyocytes in mouse hearts after I/R.

Given that DAT has been reported as an antioxidant,^[^ ^25^ ^]^ we investigated whether DAT treatment confers prosurvival effect on cardiomyocytes through antioxidant mechanisms. GSEA analysis revealed that the term positive regulation of reactive oxygen species (ROS) metabolic process was upregulated in I/R mouse hearts but was mitigated by DAT treatment (Figure S4, Supporting Information). In concert, we detected significantly increased percentages of dihydroethidium (DHE)‐positive cardiomyocytes in I/R mouse hearts at 1 d after the surgery, and these increases were significantly mitigated by DAT treatment (Figure 4F). Likewise, we observed that DAT treatment did not affect baseline conditions, but it led to a decrease in DHE fluorescence intensity in NRVMs subjected to OGD/R, indicating reduced ROS levels (Figure 4G).

Further, in line with previous reports on DAT's antioxidant effects via the regulation of the NADP^+^/NADPH ratio,^[^ ^25^ ^]^ we examined this ratio in the cells and found that DAT treatment resulted in a decrease in the NADP^+^/NADPH ratio in NRVMs subjected to OGD/R (Figure 4H). To determine if the modulation of NADP^+^/NADPH ratio is crucial for DAT's antioxidant effects in reducing cardiomyocyte death, we treated the cells with extra oxidized NADP⁺ in conjunction with DAT treatment before OGD/R exposure and showed that oxidized NADP⁺ abolished the prosurvival effect of DAT on cardiomyocytes against OGD/R (Figure 4I,J). Taken together, these data indicate that DAT attenuates cardiomyocyte death by modulating the NADP^+^/NADPH ratio against I/R.

### 2.5 DAT Suppressed the Proinflammatory Activities of Macrophages

In addition to the prosurvival effects of DAT treatment on cardiomyocytes, based on the results of DAT‐downregulated proinflammatory pathways by RNA sequencing analysis and immunohistochemical analysis of IL‐1β and TNF‐α expression showing that prophylactic DAT treatment alleviates cardiac inflammation following I/R, we proceeded to investigate the impact of DAT on immune cell populations in mouse hearts at 3 d and 1 week after the surgery. We conducted flow cytometry (FACS) analysis on single‐cell suspensions from mouse hearts after the surgery, focusing on CD45^+^ immune cells such as macrophages, neutrophils, B cells, and T cells. Our findings revealed that DAT treatment did not significantly alter the number of any immune cell types tested in mouse hearts at 3 d or 1 week after the surgery (Figure S5A,B, Supporting Information).

**Figure 5:**
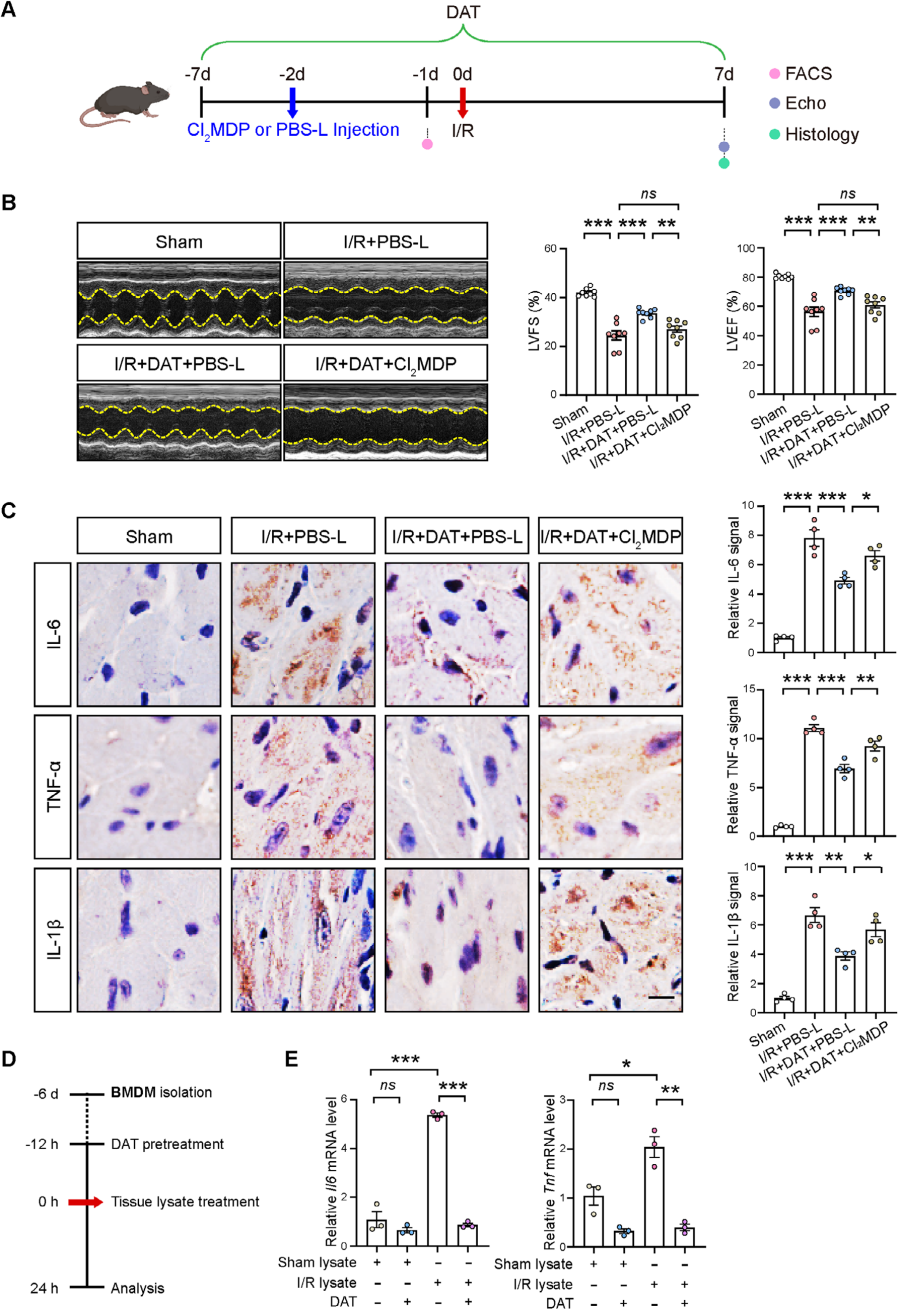
DAT suppressed the proinflammatory activities of macrophages. A) Schematics of the experiment in mice with macrophage depletion. Clodronate liposomes (Cl_2_MDP) were intravenously injected to deplete macrophages in vivo before the mice were subjected to I/R surgery. PBS liposomes (PBS‐L) was used as control for Cl_2_MDP. B) Representative echocardiographic images of mouse hearts from Sham, PBS‐L‐injected I/R (I/R+PBS‐L), DAT‐treated PBS‐L‐injected I/R (I/R+DAT+PBS‐L), and DAT‐treated Cl_2_MDP‐injected I/R (I/R+DAT+Cl_2_MDP) groups at 1 week after I/R (left panel). Quantitative data on LVFS and LVEF are shown to the right. *n =* 8 for each group. C) Representative images and quantification of IL‐6 (left panel), TNF‐α (middle), and IL‐1β (right panel) signals by immunostaining at 1 week after I/R. Scale bar, 10 µm. *n =* 4 for each group. D) Schematic diagram illustrating the in vitro experiments with bone marrow‐derived macrophages (BMDMs) stimulated with lysates from injured cardiac tissues of I/R mouse hearts 1 d after the surgery (I/R lysate), with lysates from the same region of cardiac tissues of mouse hearts subjected to Sham surgery (Sham lysate) as controls in the presence or absence of DAT pretreatment at 100 µm. E) qRT‐PCR analysis showing the mRNA levels of *Il6* and *Tnf*. *n =* 3 for each group. Quantitative data are presented as mean ± SEM. Groups were compared using B,C) one‐way ANOVA or E) two‐way ANOVA. *ns*, not significant; *p < 0.05; **p < 0.01; ***p < 0.001.

Given the RNA sequencing data that showed DAT's downregulation of inflammatory pathways, including the Fc gamma receptor‐mediated phagocytosis process where macrophages are major cell types,^[^ ^31^, ^32^ ^]^ we hypothesized that DAT's modulation of inflammatory responses following I/R might be primarily through the modulation of macrophage activity. To test this, we employed clodronate liposomes for in vivo macrophage depletion,^[^ ^33^, ^34^ ^]^ which was confirmed via FACS analysis at 1 d postinjection (Figure S5C, Supporting Information). Subsequently, mice underwent I/R surgery followed by subsequent analyses (Figure **5**A). Our results demonstrated that macrophage depletion blunted the cardioprotective effects of DAT treatment (Figure 5B). Additionally, immunohistochemical analysis revealed that macrophage depletion blunted the suppressive effects of DAT treatment on the production of proinflammatory cytokines IL‐6, TNF‐α, and IL‐1β in I/R‐injured hearts (Figure 5C). Collectively, our data indicate that DAT exerts its anti‐inflammatory effects, at least in part, through the modulation of macrophage activity.

We further explored the role of DAT in modulating macrophage activity. To this end, we conducted an in vitro experiment using cultured bone marrow‐derived macrophages (BMDMs), which were treated with lysates from ischemia‐reperfused cardiac tissues of I/R mouse hearts 1 d after the surgery (I/R lysate), with lysates from the same region of cardiac tissues of mouse hearts subjected to Sham surgery (Sham lysate) as controls, in the presence or absence of DAT (Figure 5D). Compared to Sham lysate, I/R lysate significantly increased messenger RNA (mRNA) levels of proinflammatory cytokines in BMDMs, including interleukin 6 (*Il6*) and tumor necrosis factor (*Tnf*), both of which were significantly reduced by DAT treatment (Figure 5E). Taken together, these results suggest that DAT suppresses the proinflammatory activities of macrophages in mouse hearts after I/R.

### 2.6 DAT Inhibited Proinflammatory IL‐6 Production by Macrophages

**Figure 6:**
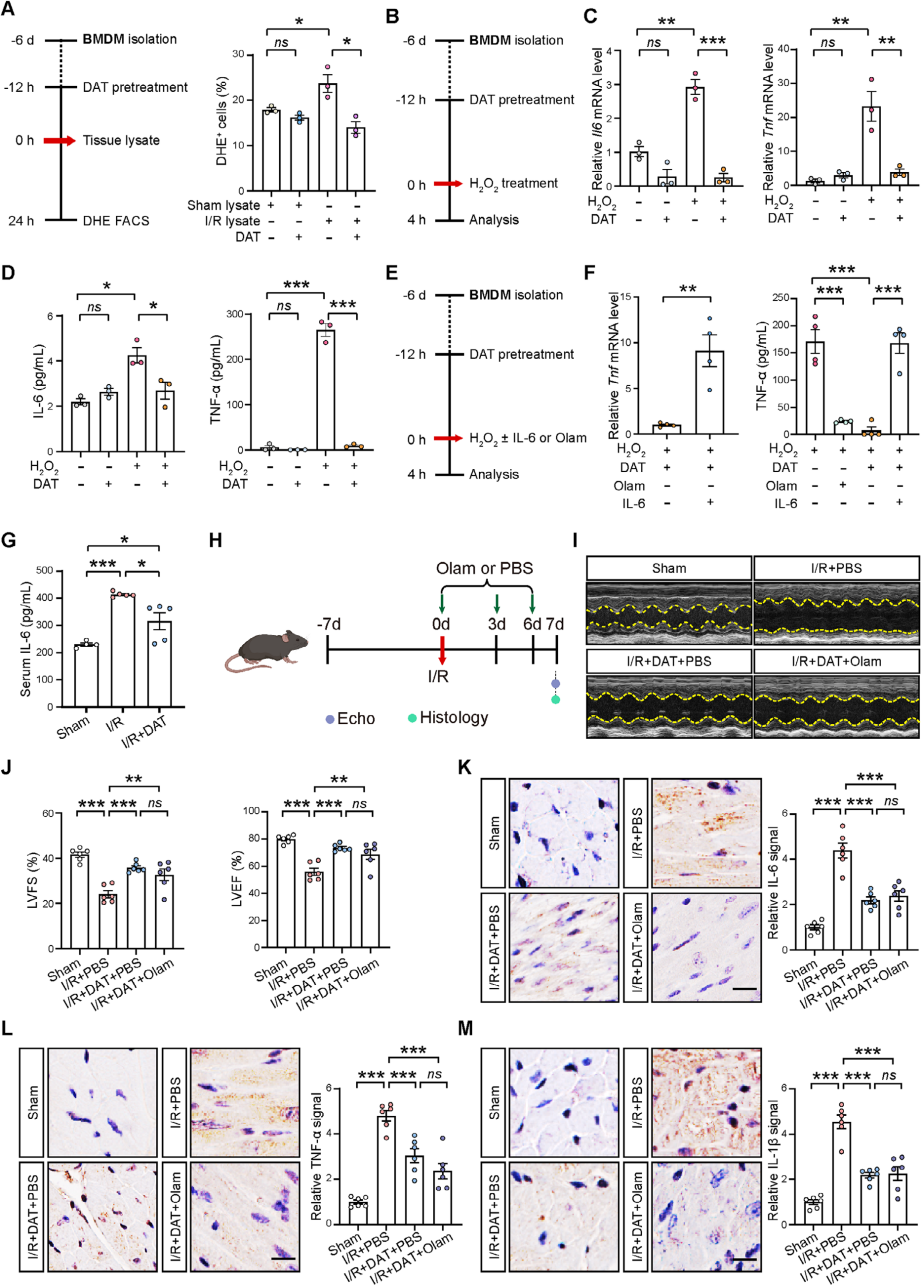
DAT inhibited proinflammatory IL‐6 production by macrophages. A) Schematic diagram (left panel) and flow cytometry analysis of DHE‐positive BMDMs stimulated with Sham lysate and I/R lysate for 24 h with or without DAT pretreatment at 100 µm (right panel). *n =* 3 for each group. B) Schematic diagram illustrating the in vitro experiments with BMDMs stimulated with 4 h H_2_O_2_ in the presence or absence of DAT pretreatment at 100 µm. C) qRT‐PCR analysis showing the mRNA levels of *Il6* and *Tnf* in BMDMs. *n =* 3 for each group. D) ELISA showing the secretion of IL‐6 and TNF‐α by BMDMs. *n =* 3 for each group. E) Schematic diagram illustrating the in vitro experiments with H_2_O_2_‐stimulated BMDMs with or without IL‐6 supplementation or Olamkicept treatment (Olam) (IL‐6 receptor antagonist) in the presence or absence of DAT‐pretreatment (100 µm). F) qRT‐PCR analysis of *Tnf* mRNA levels in BMDMs (left panel) and ELISA of TNF‐α secretion by BMDMs (right panel). *n =* 4 for each group. G) Serum IL‐6 levels in mice from the Sham, I/R, and DAT‐treated I/R (I/R+DAT) groups at 1 d after the surgery. *n = *4 for Sham group; *n = *5 for I/R and I/R+DAT groups. H) Schematic diagram illustrating the experiments in mice from Sham, PBS‐injected I/R (I/R+PBS), DAT‐treated PBS‐injected I/R (I/R+DAT+PBS), and DAT‐treated Olamkicept‐injected I/R (I/R+DAT+Olam) groups at 1 week after the surgery. I) Representative echocardiographic images of mice from Sham, I/R+PBS, I/R+DAT+PBS, and I/R+DAT+Olam groups at 1 week after the surgery. J) Quantitative data of LVFS and LVEF. *n* = 6 for each group. K–M) Representative images and quantification of K) IL‐6, L) TNF‐α, M) and IL‐1β signals by immunostaining at 1 week after I/R. *n* = 6 for each group. Scale bar, 10 µm. Quantitative data are presented as mean ± SEM. Groups were compared using G,J,K,L,M) one‐way ANOVA, A, C, D, and F‐right panel) two‐way ANOVA or F‐left panel) Student's *t*‐test. *ns*, not significant; *p < 0.05; **p < 0.01; ***p < 0.001.

The antioxidant properties of DAT have been associated with its modulation of immune response.^[^ ^25^ ^]^ Accordingly, we examined the ROS levels in BMDMs stimulated by Sham lysate and I/R lysate, with or without DAT by DHE staining. Our results indicated that I/R lysate significantly increased DHE‐positive cells compared to Sham lysate, an effect that was significantly reduced by DAT treatment (**Figure** **6**A). To determine if the antioxidant properties of DAT are crucial for its anti‐inflammatory abilities in macrophages as demonstrated above, we treated BMDMs with oxidized NADP^+^ in conjunction with I/R lysate and DAT. Our analysis revealed that oxidized NADP^+^ abolished the anti‐inflammatory effects of DAT on BMDMs stimulated by I/R lysates (Figure S6A, Supporting Information). Additionally, BMDMs were treated with H_2_O_2_ for 4 h in the presence or absence of DAT (Figure 6B). Quantitative polymerase chain reaction (qPCR) analysis revealed that the expression of *Il6* and *Tnf* mRNA was significantly increased in H_2_O_2_‐stimulated BMDMs, and this increase was effectively reduced by DAT treatment (Figure 6C). Consistent with these findings, the secretion of IL‐6 and TNF‐α proteins was also found to be significantly increased in H_2_O_2_‐stimulated BMDMs, which was also significantly reduced by DAT treatment (Figure 6D). These findings suggest that DAT suppressed I/R‐induced proinflammatory cytokine production in macrophages in a redox‐dependent manner.

DAT has been reported to reduce IL‐6 production in macrophages upon lipopolysaccharide (LPS) stimulation.^[^ ^25^ ^]^ Accordingly, to determine whether the redox‐dependent regulation of IL‐6 expression by DAT is crucial for its anti‐inflammatory effect against myocardial I/R injury, we modulated IL‐6 activity in H_2_O_2_‐stimulated BMDMs via IL‐6 supplementation and treatment with an IL‐6 receptor antagonist, Olamkicept,^[^ ^35^, ^36^ ^]^ to assess the subsequent effects on the expression of other proinflammatory cytokines by these BMDMs (Figure 6E). We observed that Olamkicept treatment resulted in a significant reduction in TNF‐α secretion in H_2_O_2_‐stimulated BMDMs, whereas IL‐6 supplementation counteracted the suppressive effect of DAT on *Tnf* expression and TNF‐α secretion in H_2_O_2_‐stimulated BMDMs (Figure 6F), highlighting IL‐6 as an upstream target for DAT's anti‐inflammatory action on macrophages. Consistent with these in vitro results, serum IL‐6 levels in mice at 1 d after the surgery by enzyme‐linked immunosorbent assay (ELISA) revealed that DAT treatment significantly reduced I/R‐induced increases in IL‐6 levels (Figure 6G). Lastly, we treated mice subjected to I/R surgery with either DAT alone, Olamkicept alone, or a combination of both (Figure 6H). Although both DAT and Olamkicept individually alleviated cardiac dysfunction after I/R, the concurrent use of Olamkicept did not augment the cardioprotective effects of DAT, as evidenced by cardiac function assessments via echocardiogram (Figure 6I,J and Figure S6B, Supporting Information) and the expression of proinflammatory cytokines in mouse hearts by immunohistochemical analysis (Figure 6K–M). This absence of synergistic enhancement suggests that both agents might be converging on the same anti‐inflammatory pathway, notably the suppression of proinflammatory cytokine IL‐6 in macrophages. In summary, these data indicate that DAT mitigates cardiac inflammation by decreasing macrophage‐derived IL‐6 production in mouse hearts after I/R.

## 3 Discussion

In this study, we uncovered the cardioprotective potential of *F. plautii* in mitigating myocardial I/R injury. Our findings demonstrate that prophylactic *F. plautii* gavage significantly enhanced cardiac function and reduced heart injury. Notably, its metabolite, DAT, was found to increase in serum and cardiac tissues following *F. plautii* gavage and recapitulate the cardioprotective effects against myocardial I/R injury. Transcriptomic analysis revealed that DAT ameliorated cardiac cell death and inflammation induced by I/R. Mechanistic studies further indicated that DAT promoted cardiomyocyte survival via the modulation of the NADP^+^/NADPH ratio. Additionally, DAT attenuated I/R‐induced cardiac inflammation via the suppression of macrophage proinflammatory activities by suppressing IL‐6 production. Collectively, our findings provide a therapeutic strategy through the prophylactic use of *F. plautii* or its metabolite DAT against myocardial I/R injury.

Previous studies have identified several probiotics and their metabolites can alleviate myocardial I/R injury through various mechanisms.^[^ ^5^, ^6^ ^]^ For instance, our previous research indicates that *Lactobacillus reuteri* and its metabolite GABA reduced cardiac inflammation by inhibiting macrophage lysosomal leakage and inflammasome activation, thereby suppressing macrophage M1 polarization and decreasing the number of proinflammatory macrophages in the heart upon myocardial I/R injury.^[^ ^6^ ^]^ Additionally, *Bifidobacterium infantis* and its metabolite inosine attenuate cardiac inflammation through A2A receptor (A2AR) activation in immune cells including natural killer cells and dendritic cells, while also suppressing cardiac cell death by serving as an alternative carbon source for adenosine triphosphate (ATP) production upon myocardial I/R injury.^[^ ^5^ ^]^ Here, we investigated the potential cardioprotective effects of *F. plautii* and its metabolite DAT against myocardial I/R injury. Our findings demonstrate that DAT enhances cardiomyocyte survival through antioxidant actions mediated by modulation of the NADP^+^/NADPH ratio, a mechanism that has not been previously explored in our earlier work or in other studies targeting cardiovascular diseases with microbiota‐derived interventions.^[^ ^5^, ^6^, ^22^ ^]^ Additionally, DAT specifically suppresses macrophage proinflammatory activities by reducing IL‐6 production in a redox‐dependent manner, without altering macrophage numbers, highlighting a redox‐dependent mechanism for immune modulation in the context of myocardial I/R injury. This study expands the potential of gut microbiota‐based therapeutic strategies beyond previously reported strains and mechanisms for mitigating myocardial I/R injury. It highlights the potential for personalized therapeutic approaches using specific cardioprotective strains or combinations thereof, tailored to individual gut microbiota composition. Future research is needed to determine the most effective and practical applications of *F. plautii* and DAT, either alone or in combination with other strains, for treating myocardial I/R injury.

Myocardial I/R injuries are accompanied by inflammatory response, which exacerbates infarct size and subsequent cardiac remodeling.^[^ ^25^ ^]^ As a gut microbiota‐derived metabolite, DAT exhibits anti‐inflammatory properties capable of modulating both local and systemic immune responses.^[^ ^24^, ^26^ ^]^ Previous studies have shown that DAT enhances host defense against influenza by via the augmentation of type I interferon signaling and the reduction of lung immunopathology.^[^ ^26^ ^]^ Additionally, DAT has been reported to enhance the antimicrobial functions of macrophages while reducing IL‐6 production upon LPS stimulation.^[^ ^25^ ^]^ Here, our findings indicate that DAT mitigates myocardial I/R injury by suppressing macrophage IL‐6 production in a redox‐dependent manner, aligning with prior research on DAT's anti‐inflammatory effects in infectious conditions and extends its regulatory role to noninfectious cardiac diseases. Notably, recent studies have also highlighted that various bioactive compounds, including flavonoids such as quercetin, exert cardioprotective effects by the suppression of inflammatory response, often resulting in the promotion of cardiac cell survival^[^ ^37^, ^38^, ^39^ ^]^ via the crosstalk between macrophages and cardiomyocytes, raising the possibility that that the prosurvival effects of DAT on cardiomyocytes may be partly attributed to its anti‐inflammatory properties via the macrophage‐cardiomyocyte crosstalk.

Due to the adult heart's limited regenerative potential, identifying new strategies for promoting the survival of cardiomyocytes following I/R injury represents a goal of cardiac biology.^[^ ^40^, ^41^ ^]^ ROS contribute to myocardial injury and death during I/R injury through various molecular mechanisms, and strategies reducing ROS levels have been employed to attenuate myocardial damage.^[^ ^42^, ^43^ ^]^ DAT as a redox‐active microbial metabolite has been primarily associated with immune modulation, often via its antioxidant actions.^[^ ^25^ ^]^ Yet, there is no direct evidence linking it to the direct promotion of (cardiac) cell survival. Our study demonstrates that DAT directly enhanced cardiomyocyte survival mediated through its antioxidant properties, thereby reinforcing the therapeutic potential of employing *F. plautii* and DAT in combating myocardial I/R injury.

In summary, our study provides evidence that prophylactic administration of *F. plautii* mitigates myocardial I/R injury and highlights its metabolite, DAT, as a cardioprotective agent with both prosurvival and anti‐inflammatory properties. This work not only provides a scientific basis for the translational application of *F. plautii* and DAT in the treatment of myocardial I/R injury but also points to their broader implications in treating a range of cardiovascular diseases and noncardiac disorders that are characterized by cell death and inflammatory responses.

## 4 Experimental Section

### Mouse Husbandry and Experiments

All the C57BL/6J mice used in this study were obtained from SPF (Beijing) Biotechnology Co., Ltd. (Beijing, China). All the animal procedures were approved by the Institutional Care and Ethical Committee of the Institute of Zoology, Chinese Academy of Sciences (permit number: IOZ‐IACUC‐2023‐249). Upon purchase, mice were housed in ventilated cages and fed an autoclaved diet under a strict 12 h light cycle in an SPF animal facility. Euthanasia was performed via isoflurane inhalation, followed by cervical dislocation.

a) *Flavonifractor Plautii* Gavage

To deplete the gastrointestinal microbiota of mice, a cocktail of antibiotics was prepared and administered ad libitum in drinking water for 7 d. The cocktail consisted of vancomycin (0.125 mg mL^−1^ d^−1^) (1404‐94‐9, Sigma‐Aldrich, USA), neomycin (0.25 mg mL^−1^ d^−1^) (1405‐10‐3, Macklin, China), metronidazole (0.25 mg mL^−1^ d^−1^) (443‐48‐1, Solarbio, China), and ampicillin (0.25 mg mL^−1^ d^−1^) (7177‐48‐2, Solarbio, China), with aspartame (0.1 mg mL^−1^ d^−1^) (A801109, Macklin, China) added to the mixture to improve palatability.^[^ ^44^ ^]^

*F. plautii* strain was obtained from the American Type Culture Collection (ATCC 49531) and stored at −80 °C in 20% glycerol. Prior to the experiments, the bacteria were propagated twice in de Man, Rogosa and Sharpe (MRS) broth (Hopebio, China) overnight at 37 °C. After propagation, cell pellets were collected by centrifugation at 8000 × *g* for 10 min at 4 °C and resuspended in PBS to achieve a concentration of 2.5 × 10^9^ CFU mL^−1^. Each mouse was then administered a daily gavage of 200 µL of the bacterial suspension, delivering a dose of 5 × 10^8^ CFU per mouse.

b) Ischemia/Reperfusion Studies In Vivo

Mice were anesthetized with 2% isoflurane, and their chest cavities were opened at the fourth intercostal space. The pericardium was subsequently removed to expose the heart. The LAD coronary artery was then ligated using a silk ligature to induce ischemia for 40 min, and the ligature was then released to allow reperfusion of the ischemic area.

c) DAT Administration In Vivo

Mice were provided with drinking water supplemented with DAT at a concentration of 20 mg mL^−1^, which resulted in serum DAT levels comparable to those achieved through the aforementioned gavage administration of *F. plautii*. DAT‐supplemented water was freshly prepared and replaced every 3 d throughout the experiment.

d) Olamkicept Administration In Vivo

Mice subjected to I/R surgery received intraperitoneal injections of 200 µL of Olamkicept solution (1 mg kg^−1^) (T73209, TargetMol, USA) or PBS at 5 min before reperfusion, 3 d after reperfusion, and 6 d after reperfusion.

e) Macrophage Depletion

Mice were intravenously injected with 200 µL of clodronate liposomes (Liposoma, the Netherlands) 2 d prior to I/R surgery. Macrophage depletion was validated through FACS analysis of circulating monocytes.

f) Echocardiography

To assess the cardiac function of mice from different groups, a Visual Sonics Vevo 3100 imaging system (FUJIFILM VisualSonics) was utilized to conduct transthoracic echocardiography with a 30 MHz transducer (MX400). 2D M‐mode traces were captured at the level of the papillary muscle of each mouse that was anesthetized with a mixture of 1.5% isoflurane and 100% oxygen. Parameters such as LVFS, LVEF, LVEDD, and LVESD were measured using an ImageJ software (ImageJ2, Fiji) from three consecutive cardiac cycles.

### Quantification of 16S rDNA

qPCR was performed on genomic DNA isolated from feces samples by using the DNeasy PowerSoil Pro Kits (Qiagen, Germany). After extraction, all DNA concentrations were measured with the Qubit dsDNA High Sensitivity Assay Kit (Thermo Fisher Scientific, USA). Bacteria‐specific 16S primers (Fw: 5′‐TCCTACGGGAGGCAGCAGT‐3′ and Re: 5′‐GGACTACCAGGGTATCTAATCCTGTT‐3′) and *F. plautii*‐specific primers (Fw: 5′‐TGAGTAACGCGTGAGGAACC‐3′ and Re: 5′‐TCGTCGGGTACCGTCATTTG‐3′) were used to measure the absolute abundance of bacteria 16S rDNA and *F. plautii* gene copies, respectively. Standard curves were generated by using 1:5 dilutions of the stock of *E. coli* and *F. plautii* 16S rDNA amplicons. All qPCR reactions were carried using Taq Pro universal synergetic binding reagent (SYBR) qPCR Master Mix (Vazyme #Q712) and QuantStudio 7 Flex Real‐Time PCR Systems. The abundance of total bacteria or *F. plautii* was then calculated based on the cycle threshold (Ct) values obtained from the real‐time PCR, in conjunction with the standard curve.^[^ ^22^ ^]^

### Histological Analysis of Mouse Heart

For the preparation of paraffin‐embedded sections, after fixation in 4% paraformaldehyde (DF0131, Leagene Biotechnology), heart tissues underwent dehydration through graded alcohols, were embedded in paraffin wax, and sectioned at 5 µm thickness. Subsequently, the sections were deparaffinized with xylene and rehydrated via a sequential ethanol process. For the preparation of frozen sections, fresh heart tissues were immediately placed in optimal cutting temperature compound and rapidly frozen in liquid nitrogen. The frozen tissues were then mounted onto a cryostat microtome and sectioned at 10 µm thickness. The sections were collected on superfrost slides and stored at −80 °C until further use. Before staining, the sections were air‐dried for 30 min and fixed with cold acetone for 10 min at −20 °C.

a) Quantification of Cardiac Fibrosis by Picrosirius Red (PSR) Staining

Cardiac fibrosis was measured via PSR staining. Rehydrated sections were incubated in Picrosirius red dye (DC0041, Leagene Biotechnology, China) for 1 h at room temperature. Following rinsing in running water, sections were dehydrated through an ethanol series and xylene, and mounted with neutral balsam (N116470, Aladdin, China).

b) Immunohistochemistry

Rehydrated sections were treated with 3% H_2_O_2_ for 10 min at room temperature, followed by the treatment with sodium citrate buffer for antigen retrieval. Sections were then blocked with a blocking buffer containing 5% bovine serum (A8020, Solarbio, China) for 1 h at room temperature, followed by the overnight incubation at 4 °C with primary antibodies against IL‐1β (1:200, 16806‐1‐AP, Proteintech, China), TNF‐α (1:200, A0277, Abclonal, China), and IL‐6 (1:200, 26404‐1‐AP, Proteintech, China). The next day, after three 10 min washes in PBS, sections were incubated with a biotinylated goat antirabbit secondary antibody (SP‐0022 SP Kit, Bioss, China) for 1 h, followed by streptavidin horseradish peroxidase (SP‐0022 SP Kit, Bioss, China) for 30 min at room temperature. Visualization was achieved using 3,3′‐diaminobenzidine tetrahydrochloride (8059, Cell Signaling, USA), and sections were counterstained with hematoxylin before dehydration and mounting with neutral balsam (N116470, Aladdin, China). Images were captured with an Olympus microscope IX73 and quantified using an ImageJ software (ImageJ2, Fiji).

c) 2,3,5‐Triphenyltetrazolium Chloride (TTC) Staining

Mice were perfused with saline and euthanized 24 h after I/R or sham surgery. The hearts were then collected and frozen at −80 °C overnight. Subsequently, the hearts were then evenly sectioned from the ligation site to the apex at 1 mm thickness and stained with 1% 2,3,5‐triphenyltetrazolium chloride (TTC) at 37 °C for 15 min. Sections were fixed in 4% w/v paraformaldehyde for 24 h. The infarct size was determined by dividing the volume of the nonstained infarcted region by the volume of the left ventricle measured from the ligation site to the apex using a weight‐based method with modifications based on a previously published approach.^[^ ^45^ ^]^

d) TUNEL Staining

Cardiac cell apoptosis was evaluated using a TUNEL Apoptosis Detection Kit (A113‐03, Vazyme, China) according to the manufacturer's instructions. Briefly, rehydrated sections were washed three times with PBS for 5 min each, fixed with 4% formalin for 15 min at room temperature, and incubated with the TUNEL working solution at 37 °C for 1 h. After three 5 min washes with PBS, sections were mounted with a 2‐(4‐amidinophenyl)‐6‐indolecarbamidine dihydrochloride (DAPI)‐containing solution (C0065, Solarbio, China). Images were captured with an Olympus microscope IX73 and the proportion of TUNEL‐positive cells was quantified by calculating the ratio of TUNEL‐positive nuclei to the total number of nuclei using an ImageJ software (ImageJ2, Fiji).

e) DHE Staining

ROS levels were assessed by DHE staining on frozen sections from heart samples fresh collected the day before the staining according to a previously published protocol.^[^ ^46^ ^]^ Frozen sections were retrieved from the −80 °C freezer and transferred into precooled methanol for fixation at 4 °C for 15 min. After fixation, the sections were washed three times with PBS at room temperature for 5 min each. A working solution of DHE (C0065, Solarbio, China) was prepared at a final concentration of 5 µm in PBS and stored in the dark and was evenly applied to the sections, ensuring complete coverage. The sections were then stained in the dark at room temperature for 30 min. Following staining, the sections were washed again three times with PBS at room temperature for 5 min each. Finally, a 50% glycerol solution containing 1 µm DAPI (C0065, Solarbio, China) was applied to the sections, and a coverslip was carefully placed to avoid air bubbles. Imaging was performed using an Andor Dragonfly 505 confocal spinning disk system and DHE‐positive nuclei were quantified using ImageJ software (ImageJ2, Fiji).

### Measurement of DAT Concentration by High‐Performance Liquid Chromatography‐Mass Spectrometry (HPLC‐MS)

DAT concentration was determined using HPLC‐MS as previously described.^[^ ^47^ ^]^ For culture medium, fresh culture medium or the culture medium of *Flavonifractor plautii* was collected after centrifugation at 12 000 rpm for 15 min and the supernatant was collected for further analysis. For serum, 30 µL of methanol (38460, Sigma, USA) was added to 15 µL of serum, and the resultant mixture was at 12 000 rpm for 15 min. The supernatant was collected for further analysis. For heart tissue, appropriate amounts of heart tissues were accurately weighed, placed in 2 mL centrifuge tubes, and 1.5 mL of ice‐cold methanol was added for grinding for 30 s. The samples were then subjected to ultrasonication on ice at 300 W and 40 kHz for 15 min. After centrifugation at 12 000 rpm for 15 min, the supernatant was collected and dried using centrifugal evaporation. The dried samples were stored at −80 °C. Prior to analysis, the residue was reconstituted with 80% methanol, vortexed for 3 min, and centrifuged at 12 000 rpm for 20 min. The supernatant was collected for further analysis.

The samples were analyzed using an Agilent 1290 Infinity II LC system coupled to an Agilent 6495 triple quadrupole LC/MS (Agilent 1290‐6495, Agilent Technologies, USA). Separation was achieved using an Agilent Poroshell Pentafluorophenyl (PFP) HPLC Column (2.1 × 150 mm, USA). Mobile phase A was 0.1% formic acid in water, and mobile phase B was 0.1% formic acid (5.43804.0250, Merck Millipore, USA) in 95% acetonitrile (34851, Merck Millipore, USA). The flow rate was set to 0.25 mL min^−1^, starting at 0% B held constant for 3 min, then linearly increased to 50% over 5 min, followed by a linear increase to 95% B over 1 min, and held at 100% B for the next 3 min. Mobile phase B was then brought back down to 0% over 0.5 min and held at 0% for re‐equilibration for 2.5 min. Transitions were monitored in negative mode, with the transition for DAT being m/z 165.17 to m/z 93.1.

### Primary Cells and Cell Lines

a) Isolation, Culture, and Treatments of Murine Bone Marrow‐Derived Macrophages (BMDMs)

BMDMs were isolated and cultured as previously reported.^[^ ^48^, ^49^ ^]^ Briefly, bone marrow was harvested by flushing Dulbecco's modified Eagle's medium (DMEM) (C3111‐0500, Biological Industries, Israel) through the femur, tibia, and hip bones of mice using a 25‐gauge needle. The bone marrow was then resuspended in red cell lysis buffer (TBD Science, China) for 3 min, followed by centrifugation at 1700 rpm for 5 min at 4 °C. The pellet was resuspended and passed through a 40 µm cell strainer. Cells were plated at a density of 1 × 10^6^ cells mL^−1^ in DMEM supplemented with 10% fetal bovine serum (FBS, C04001500, Biological Industries) and 1% antibiotics (100 U mL^−1^ penicillin and 100 µg mL^−1^ streptomycin; 030311B, Biological Industries), containing macrophage colony‐stimulating factor (M‐CSF) (50 ng mL^−1^, STEMCELL Technologies, Canada) and maintained at 37 °C with 5% CO_2_ for 5 d. On day 6, BMDMs were treated with 50 ng mL^−1^ tissue lysates derived from mouse hearts that had undergone either sham surgery or I/R surgery at 24 h prior to sampling. For DAT (HY‐W015346, MedChemExpress, China) treatment, BMDMs were pretreated with a final concentration of 100 µm DAT for 12 h before being stimulated with tissue lysates. The concentration was determined based on the serum DAT levels observed in *F. plautii*‐gavaged mice and DAT‐treated mice, which were measured at ≈6 µm, equivalent to 36.33 µm in serum. Given that DAT was continuously produced by *F. plautii* in *F. plautii*‐gavaged mice and supplemented through drinking water in DAT‐treated mice in vivo, a threefold concentration (100 µm) was chosen in vitro to account for the sustained and dynamic production of DAT in the in vivo setting.

b) Isolation, Culture, and Treatments of Neonatal Rat Ventricular Cardiomyocytes (NRVMs)

NRVMs were isolated and cultured as previously reported.^[^ ^50^, ^51^ ^]^ The ventricles of neonatal (P0‐1) Sprague–Dawley rat hearts are carefully separated from the atria and cut into small pieces in cold Hank's balanced salt solution (HBSS) (88284, Thermo Fisher Scientific, USA). These tissue pieces were subjected to enzymatic digestion using a combination of trypsin (1 mg mL^−1^, 27250018, Thermo Fisher Scientific, USA) and collagenase II (0.8 mg mL^−1^, LS004176, Worthington, USA) in HBSS. Physical stirring during this step further facilitated the dissociation of cells from the tissue matrix. Subsequently, the collected heart cells were filtered through a 100 µm cell strainer (352360, Corning, USA). The filtered cells were then centrifuged and resuspended in DMEM (06‐1055‐57‐1A, Biological Industries, Israel) containing 10% fetal bovine serum (10099141, Gibco, USA) and 1% penicillin and streptomycin (03‐031‐1B, Biological Industries, Israel). The resuspended cells were plated onto 100 mm plastic dishes for 2 h to allow fibroblasts to adhere. The supernatant, containing mostly NRVMs, was then collected and plated onto 96‐well or 24‐well plates as appropriate.

c) Culture and Treatments of Cell Lines

Mouse NIH3T3 fibroblast cell line (BNCC100843, BeNa Culture Collection, China) and mouse cardiac endothelial cell line MCEC (MCECs, BeNa Culture Collection, China) were maintained in DMEM (C3111‐0500, Biological Industries, Israel) with 10% FBS (C04001500, Biological Industries, Israel) and 1% antibiotics (100 U mL^−1^ penicillin and 100 µg mL^−1^ streptomycin; 030311B, Biological Industries, Israel). All these cells were incubated in a humidified 37 °C incubator (3111, Thermo Fisher Scientific, USA) with 5% CO_2_.

### RNA Extraction and Quantitative Real‐Time Polymerase Chain Reaction (qRT‐PCR)

Total RNA was extracted from cells or mouse heart tissues using TRIzol Reagent (15596018, Thermo Fisher Scientific, USA). Complementary cDNA was synthesized using the RevertAid Master Mix (M1631, Thermo Fisher Scientific, USA). qRT‐PCR was performed using Taq Pro universal synergetic binding reagent (SYBR) qPCR Master Mix (Vazyme #Q712). The mRNA levels of target genes were normalized to those of *18S* and *Actb* as appropriate internal controls. The sequences of the primers used for qRT‐PCR are listed below.

*Tnf* Fw: 5′‐TATGGCTCAGGGTCCAACTC‐3′;

*Tnf* Re: 5′‐CTCCCTTTGCAGAACTCAGG‐3′;

*Il6* Fw: 5′‐TTCCATCCAGTTGCCTTCTT‐3′;

*Il6* Re: 5′‐TCCACGATTTCCCAGAGAAC‐3′;

*18s* Fw: 5′‐GCTTAATTTGACTCAACACGGGA‐3′;

*18s* Re: 5′‐AGCTATCAATCTGTCAATCCTGTC‐3′;

*Actb* Fw: 5′‐TGTTACCAACTGGGACGACA‐3′;

*Actb* Re: 5′‐GGGGTGTTGAAGGTCTCAAA‐3′.

### RNA Sequencing and Data Analysis

Sequencing libraries were generated using Illumina Stranded mRNA Prep, Ligation Kit from Illumina (San Diego, CA) following the manufacturer′s instructions. Qualified libraries were sequenced on Illumina platforms using the PE150 strategy by Majorbio (Shanghai, China). R package DESeq2 (version 1.12.3) was utilized to analyze differentially expressed genes (DEGs), with simultaneous thresholds set for a fold change no less than 2 and an adjusted *p*‐value less than 0.05 (using the Benjamini‐Hochberg method).^[^ ^52^, ^53^ ^]^ Subsequently, principal component analysis (PCA) was conducted on the full set of processed detected genes. With variance stabilizing transformation of the read counts, PCA was performed using the prcomp function of R Stats Package (version 3.6.2). DEGs were further analyzed using volcano plots, heatmaps, KEGG analysis, and GSEA. Visualization of the volcano plot and histogram was achieved using R package ggplot2 (version 3.4.2). KEGG enrichment analysis was conducted with clusterProfiler (version 4.8.2) in R and visualized with ComplexHeatmap (version 2.16.0). GSEA was performed using the GSEA software (version 4.3.2).^[^ ^54^, ^55^ ^]^

### Measurement of Cytokine Levels by ELISA

Blood samples were collected from the mouse facial vein at specified time points and allowed to clot undisturbed at 4 °C for 4 h, followed by centrifugation at 1000 × *g* for 10 min. The serum was then carefully collected from the resultant supernatant for further analysis. The levels of serum TNFα (RK00027, Abclonal, China), IL‐1β (RK00006, Abclonal, China), and IL‐6 (RK00008, Abclonal, China) were quantified using respective ELISA kits according to the manufacturer's instructions.

### Flow Cytometry Analysis of Immune Cells in the Heart

Single‐cell suspensions of mouse hearts were prepared as described previously.^[^ ^56^ ^]^ Mouse hearts were injected with ethylenediaminetetraacetic acid (EDTA) buffer (containing 130 mm NaCl, 5 mm KCl, 0.5 mm NaH_2_PO_4_, 10 mm 4‐(2‐hydroxyethyl)piperazine‐1‐ethanesulfonic acid (HEPES), 10 mm Glucose, 10 mm 2,3‐butanedione monoxime (BDM), 10 mm Taurine, 5 mm EDTA). Subsequently, the inferior vena cava was opened. The aorta was then clamped and the heart was isolated and sequentially perfused with EDTA buffer, Perfusion buffer (containing 130 mm NaCl, 5 mm KCl, 0.5 mm NaH_2_PO_4_, 10 mm HEPES, 10 mm Glucose, 10 mm BDM, 10 mm Taurine, 1 mm MgCl_2_), and Collagenase buffer (containing 0.5 mg mL^−1^ Collagenase 2, LS004176, Worthington, USA; 0.5 mg mL^−1^ Collagenase 4, LS004188, Worthington, USA). Heart tissues were pulled gently into 1 mm^3^ pieces using forceps and dissociated by gentle pipetting in a dish with Collagenase buffer after the clamp was removed. Enzyme activity was halted after 1 h of digestion by adding stop buffer (Perfusion buffer supplemented with sterile FBS). Cell suspensions were filtered through 100 µm cell strainers and cardiomyocytes were allowed to gravity‐settle for 20 min. The supernatant, containing other cell types including immune cells, was collected and centrifuged at 300 × *g* for 5 min at 4 °C. The resultant cell pellets were then resuspended in 80 µL PBS for subsequent analysis.^[^ ^57^ ^]^

Cells were first treated with Fcγ‐blocking antibody antimouse CD16/32 for 10 min at 4 °C, followed by staining with monoclonal antibodies for 30 min. All immune cells were identified by gating on CD45⁺. Neutrophils were defined as CD11b⁺ Ly6G^high^, macrophages as CD11b⁺ F4/80⁺, B cells as CD11b⁻ B220⁺, and T cells as CD11b⁻ TCRβ⁺. The samples were analyzed using an FACS Canto II Cell Analyzer (BD Biosciences, NJ, USA), and the acquired data were analyzed with FlowJo software (version 10.8.1).

The antibodies used were as follows: Fcg‐blocking antibody antimouse CD16/32 (101319, Biolegend, CA, USA), BV421 antimouse CD45 (103134, Biolegend, USA), BV510 antimouse CD11b (101263, Biolegend, CA, USA), BV605 antimouse Ly6C (128036, Biolegend, CA, USA), fluorescein isothiocyanate (FITC) antimouse Ly6G (127605, Biolegend, CA, USA), phycoerythrin (PE) antimouse F4/80 (123109, Biolegend, CA, USA), allophycocyanin (APC) antimouse B220 (103212, Biolegend, CA, USA), PE/Cy7 antimouse TCRβ (109221, Biolegend, CA, USA).

### Flow Cytometry Analysis of ROS in BMDMs

BMDMs were incubated with 1.25 µm DHE (S0063, Beyotime, China) in suspension at 37 °C for 30 min to detect ROS. BMDMs were then collected by centrifugation at 300 × *g* for 5 min and washed twice with PBS to remove any unbound probes. All the samples were analyzed on an LSRFortessa X‐20 analyzer (BD Biosciences, USA). The acquired data were analyzed with FlowJo software (version 10.8.1).^[^ ^58^ ^]^

### OGD/R

For NIH3T3 cells and MCECs, cells were seeded into 96‐well plates at a density of 1 × 10⁵ cells mL^−1^ in DMEM supplemented with 10% FBS and 1% penicillin/streptomycin (Proliferation medium, PM) during the proliferation phase. For the normoxia group, cells were kept in a standard incubator with PM for 10 h. For the OGD/R group, cells were incubated in AVATAR hypoxia incubators (XCell Biosciences, USA) under conditions of 0.1% O₂, 5% CO₂, 94.9% N₂, and 1.0 Psi, using DMEM without glucose or FBS for 8 h, followed by reoxygenation for an additional 2 h in PM under normoxic conditions.

For NRVMs, cells were seeded into 96‐well plates at a density of 1 × 10⁵ cells mL^−1^ in PM. For the normoxia group, cells were kept in a standard incubator with PM for 17 h. For the OGD/R group, cells were incubated in AVATAR hypoxia incubators (XCell Biosciences, USA) under conditions of 0.1% O₂, 5% CO₂, 94.9% N₂, and 1.0 Psi, using DMEM without glucose or FBS for 14 h, followed by reoxygenation for an additional 3 h in PM under normoxic conditions.

### Cell Viability Assay

Cell viability was assessed using Alamar Blue fluorescence detection. The assays were performed in 96‐well plates. Upon removal of the culture medium and rinsing with PBS, a volume of 100 µL of Alamar Blue solution (10% in PBS, A7631, Solarbio, China) was added to each well, and the plates were incubated at 37 °C for 2 h. Following incubation, the fluorescence was measured in a new plate using a Cytation 5 fluorescence multiwell plate reader (Agilent, USA) with excitation and emission wavelengths set at 545 and 590 nm, respectively.

### Cell Death Assay

Cell death was evaluated by measuring the activity of lactate dehydrogenase (LDH) released from the cultured cells. Cells were seeded into 96‐well plates, and the supernatants were collected after induction of OGD/R. The LDH activity in the supernatants was assayed according to the manufacturer's instructions using the LDH Cytotoxicity Assay Kit (40209ES76, Yeasen, China).

### Measurement of ROS in Cultured Cells

Cells were stained with DHE to assess ROS levels, following the manufacturer's instructions. Nuclei were counterstained with Hoechst 33342. Imaging was performed using a Cytation 5 fluorescence multiwell plate reader (Agilent, USA), and the number of DHE‐positive nuclei was quantified by using an ImageJ software (ImageJ2, Fiji).

### Measurement of NADP^+^/NADPH Ratio

NADP⁺/NADPH ratio was analyzed using an NADP/NADPH Assay Kit (Solarbio, BC1100). NRVMs were lysed with an NADP/NADPH extraction buffer. The samples were then subjected to extraction using both acidic and alkaline extraction solutions to extract NADP⁺ and NADPH. The levels of NADP⁺ and NADPH were measured according to the manufacturer's instructions via the measurement of the absorbance at 570 nm using a Cytation 5 fluorescence multiwell plate reader (Agilent, USA).

### In Vitro Fibrosis Induction

NIH3T3 cells underwent serum starvation and were subsequently treated with either PBS or transforming growth factor‐β1 (TGF‐β1) at a concentration of 10 ng mL^−1^ (80116‐RNAH‐5, Sino Biological, China) to induce fibrosis under baseline and TGF‐β1‐stimulated conditions, respectively. The fibrosis marker α‐smooth muscle actin (α‐SMA) was analyzed using immunofluorescence staining. Briefly, the cells were stained with an α‐SMA primary antibody (14395‐1‐AP, Proteintech, China) and a fluorescent secondary antibody (AS053, Abclonal, China). The α‐SMA fluorescence intensity was then visualized and quantified using a Cytation 5 fluorescence multiwell plate reader (Agilent, USA).

### Wound Healing Assay

Wound healing assay was conducted by first seeding cells in six‐well plates until they reached confluence. A sterile pipette tip was then used to create a uniform wound across the cell monolayer and the cells were permitted to migrate into the wound area. Images of the wound were captured at predetermined time intervals using a Cytation 5 fluorescence multiwell plate reader (Agilent, USA). These images were used to evaluate the rate of cell migration by measuring the reduction in wound area over time by using an ImageJ software (ImageJ2, Fiji).

### Statistical Analysis

All statistical analyses were performed using GraphPad Prism 10.3 software (Prism Inc., San Diego, CA, USA). Comparisons between two groups were made using a two‐tailed unpaired Student′s *t*‐test when appropriate. For comparisons involving more than two groups, one‐way or two‐way analysis of variance (ANOVA) was conducted, followed by post hoc Tukey's test or Dunnett's test as applicable. Data are presented as individual data points or as mean ± standard error of the mean (SEM). Differences are considered statistically significant at *p* < 0.05.

## Conflict of Interest

The authors declare no conflict of interest.

## Supporting information



Supporting Information

## Data Availability

All the RNA sequencing data have been deposited in the National Center for Biotechnology Information Sequence Read Archive (SRA) under the accession number (PRJNA1213996).

## References

[1] A. Timmis , P. Vardas , N. Townsend , A. Torbica , H. Katus , D. De Smedt , C. P. Gale , A. P. Maggioni , S. E. Petersen , R. Huculeci , D. Kazakiewicz , V. de Benito Rubio , B. Ignatiuk , Z. Raisi‐Estabragh , A. Pawlak , E. Karagiannidis , R. Treskes , D. Gaita , J. F. Beltrame , A. McConnachie , I. Bardinet , I. Graham , M. Flather , P. Elliott , E. A. Mossialos , F. Weidinger , S. Achenbach , Eur. Heart J. 2022, 43, 716.35016208

[2] P. Joseph , D. Leong , M. McKee , S. S. Anand , J.‐D. Schwalm , K. Teo , A. Mente , S. Yusuf , Circ. Res. 2017, 121, 677. 28860318 10.1161/CIRCRESAHA.117.308903

[3] P. Li , J. Ge , H. Li , Nat. Rev. Cardiol. 2020, 17, 96. 31350538 10.1038/s41569-019-0235-9

[4] Q. Zhang , L. u. Wang , S. Wang , H. Cheng , L. Xu , G. Pei , Y. Wang , C. Fu , Y. Jiang , C. He , Q. Wei , Signal Transduction Targeted Ther. 2022, 7, 78. 10.1038/s41392-022-00925-z PMC8913803 35273164

[5] H. Zhang , J. Wang , J. Shen , S. Chen , H. Yuan , X. Zhang , X.u Liu , Y. Yu , X. Li , Z. Gao , Y. Wang , J. Wang , M. Song , iMeta 2024, 3, e220. 39135700 10.1002/imt2.220 PMC11316933

[6] J. Wang , H. Zhang , H. Yuan , S. Chen , Y. Yu , X. Zhang , Z. Gao , H. Du , W. Li , Y. Wang , P. Xia , J. Wang , M. Song , Adv. Sci. (Weinheim, Ger.) 2024, 11, e2307233. 10.1002/advs.202307233 PMC11095141 38487926

[7] M. Zhang , S. Lee , M. Huang , M. Tan , Life Med. 2024, 3, lnae003. 39872395 10.1093/lifemedi/lnae003 PMC11749452

[8] W. H. Tang , T. Kitai , S. L. Hazen , Circ. Res. 2017, 120, 1183. 28360349 10.1161/CIRCRESAHA.117.309715 PMC5390330

[9] Y. Yang , H. Zhang , Y. Wang , J. Xu , S. Shu , P. Wang , S. Ding , Y. Huang , L. Zheng , Y. Yang , C. Xiong , Imeta 2024, 3, e159. 38882495 10.1002/imt2.159 PMC11170974

[10] G. G. Schiattarella , A. Sannino , E. Toscano , G. Giugliano , G. Gargiulo , A. Franzone , B. Trimarco , G. Esposito , C. Perrino , Eur. Heart J. 2017, 38, 2948. 29020409 10.1093/eurheartj/ehx342

[11] T. Hu , Q. Wu , Q. Yao , K. Jiang , J. Yu , Q. Tang , Ageing Res. Rev. 2022, 81, 101706. 35932976 10.1016/j.arr.2022.101706

[12] X. F. Chen , X. Chen , X. Tang , Clin. Sci. 2020, 134, 657. 10.1042/CS20200128 32219347

[13] F. Z. Marques , E. Nelson , P.‐Y. Chu , D. Horlock , A. Fiedler , M. Ziemann , J. K. Tan , S. Kuruppu , N. W. Rajapakse , A. El‐Osta , C. R. Mackay , D. M. Kaye , Circulation 2017, 135, 964. 27927713 10.1161/CIRCULATIONAHA.116.024545

[14] W. H. W. Tang , D. Y. Li , S. L. Hazen , Nat. Rev. Cardiol. 2019, 16, 137. 30410105 10.1038/s41569-018-0108-7 PMC6377322

[15] A. T. Chen , J. Zhang , Y. Zhang , Imeta 2023, 2, e125. 38867928 10.1002/imt2.125 PMC10989798

[16] H. Liu , J. Zhuang , P. Tang , J. Li , X. Xiong , H. Deng , Curr. Atheroscler. Rep. 2020, 22, 77. 33063240 10.1007/s11883-020-00892-2

[17] J. Zhao , Q. Zhang , W. Cheng , Q. Dai , Z. Wei , M. Guo , F.u Chen , S. Qiao , J. Hu , J. Wang , H. Chen , X. Bao , D. Mu , X. Sun , B. Xu , J. Xie , Cardiovasc. Res. 2023, 119, 1390. 36715640 10.1093/cvr/cvad023 PMC10262181

[18] C. A. Lozupone , J. I. Stombaugh , J. I. Gordon , J. K. Jansson , R. Knight , Nature 2012, 489, 220. 22972295 10.1038/nature11550 PMC3577372

[19] T. Ogita , Y. Yamamoto , A. Mikami , S. Shigemori , T. Sato , T. Shimosato , Front. Immunol. 2020, 11, 379. 32184789 10.3389/fimmu.2020.00379 PMC7058663

[20] A. Mikami , T. Ogita , F.u Namai , S. Shigemori , T. Sato , T. Shimosato , Front. Nutr. 2020, 7, 610946. 33614691 10.3389/fnut.2020.610946 PMC7890079

[21] W. Li , Y. Sun , L. Dai , H. Chen , B. Yi , J. Niu , L. Wang , F. Zhang , J. Luo , K. Wang , R. Guo , L. Li , Q. Zou , Z. S. Ma , Y. Miao , BMC Microbiol. 2021, 21, 138. 33947329 10.1186/s12866-021-02201-6 PMC8097971

[22] S. Luo , Y. Zhao , S. Zhu , L. Liu , K. Cheng , B. Ye , Y. Han , J. Fan , M. Xia , Circ. Res. 2023, 132, 167. 36575982 10.1161/CIRCRESAHA.122.321975

[23] P. M. Ridker , T. F. Lüscher , Eur. Heart J. 2014, 35, 1782. 24864079 10.1093/eurheartj/ehu203 PMC4155455

[24] Y. Wei , J. Gao , Y. Kou , M. Liu , L. Meng , X. Zheng , S. Xu , M. Liang , H. Sun , Z. Liu , Y. Wang , FASEB J. 2020, 34, 16117. 33047367 10.1096/fj.201902900RR

[25] J. Zhou , J. Han , Y. Wei , Y. Wang , FASEB J. 2024, 38, e23844. 39046365 10.1096/fj.202400638R

[26] A. L. Steed , G. P. Christophi , G. E. Kaiko , L. Sun , V. M. Goodwin , U. Jain , E. Esaulova , M. N. Artyomov , D. J. Morales , M. J. Holtzman , A. C. M. Boon , D. J. Lenschow , T. S. Stappenbeck , Science 2017, 357, 498. 28774928 10.1126/science.aam5336 PMC5753406

[27] A. Braune , M. Blaut , Gut microbes 2016, 7, 216. 26963713 10.1080/19490976.2016.1158395 PMC4939924

[28] J. Su , M. Luo , N. Liang , S. Gong , W. Chen , W. Huang , Y. Tian , A. Wang , Front. Pharmacol. 2021, 12, 745061. 34504432 10.3389/fphar.2021.745061 PMC8421530

[29] B. W. Van Tassell , S. Toldo , E. Mezzaroma , A. Abbate , Circulation 2013, 128, 1910. 24146121 10.1161/CIRCULATIONAHA.113.003199 PMC3938092

[30] J. K. Pai , T. Pischon , J. Ma , J. E. Manson , S. E. Hankinson , K. Joshipura , G. C. Curhan , N. Rifai , C. C. Cannuscio , M. J. Stampfer , E. B. Rimm , N. Engl. J. Med. 2004, 351, 2599. 15602020 10.1056/NEJMoa040967

[31] C. J. Fitzer‐Attas , M. Lowry , M. T. Crowley , A. J. Finn , F. Meng , A. L. DeFranco , C. A. Lowell , J. Exp. Med. 2000, 191, 669. 10684859 10.1084/jem.191.4.669 PMC2195832

[32] Y. Hu , H. Li , W. Wang , F. Sun , C. Wu , W. Chen , Z. Liu , Nano Lett. 2023, 23, 5562. 37289965 10.1021/acs.nanolett.3c00957

[33] M. Naito , H. Nagai , S. Kawano , H. Umezu , H. Zhu , H. Moriyama , T. Yamamoto , H. Takatsuka , Y. Takei , J. Leukocyte Biol. 1996, 60, 337. 8830790 10.1002/jlb.60.3.337

[34] N. van Rooijen , A. Sanders , T. K. van den Berg , J. Immunol. Methods 1996, 193, 93. 8690935 10.1016/0022-1759(96)00056-7

[35] G. H. Waetzig , S. Rose‐John , Expert Opin. Ther. Targets 2012, 16, 225. 22324903 10.1517/14728222.2012.660307

[36] S. Schreiber , K. Aden , J. P. Bernardes , C. Conrad , F. Tran , H. Höper , V. Volk , N. Mishra , J. I. Blase , S. Nikolaus , J. Bethge , T. Kühbacher , C. Röcken , M. Chen , I. Cottingham , N. Petri , B. B. Rasmussen , J. Lokau , L. Lenk , C. Garbers , F. Feuerhake , S. Rose‐John , G. H. Waetzig , P. Rosenstiel , Gastroenterology 2021, 160, 2354. 33667488 10.1053/j.gastro.2021.02.062

[37] D. Hu , R. Li , Y. Li , M. Wang , L. Wang , S. Wang , H. Cheng , Q. Zhang , C. Fu , Z. Qian , Q. Wei , Adv. Sci. (Weinheim, Ger.) 2024, 11, e2308910. 10.1002/advs.202308910 PMC11151042 38582507

[38] X. Ai , W. Lu , K. Zeng , C. Li , Y. Jiang , P. Tu , Anal. Chem. 2018, 90, 4485. 29533659 10.1021/acs.analchem.7b04833

[39] K. Zhang , Y. Wang , S. Chen , J. Mao , Y. Jin , H. Ye , Y. Zhang , X. Liu , C. Gong , X. Cheng , X. Huang , A. Hoeft , Q. Chen , X. Li , X. Fang , Nat. Metab. 2023, 5, 129. 36635449 10.1038/s42255-022-00715-5 PMC9886554

[40] J. Zhang , R. Bolli , D. J. Garry , E. Marbán , P. Menasché , W.‐H. Zimmermann , T. J. Kamp , J. C. Wu , V. J. Dzau , J. Am. Coll. Cardiol. 2021, 78, 2092. 34794691 10.1016/j.jacc.2021.09.019 PMC9116459

[41] G. Heusch , Nat. Rev. Cardiol. 2020, 17, 773. 32620851 10.1038/s41569-020-0403-y

[42] H. Bugger , K. Pfeil , Biochim. Biophys. Acta, Mol. Basis Dis. 2020, 1866, 165768. 32173461 10.1016/j.bbadis.2020.165768

[43] M. Hori , K. Nishida , Cardiovasc. Res. 2009, 81, 457. 19047340 10.1093/cvr/cvn335

[44] T. W. H. Tang , H.‐C. Chen , C.‐Y. Chen , C. Y. T. Yen , C.‐J. Lin , R. P. Prajnamitra , L.‐L. Chen , S.‐C. Ruan , J.‐H. Lin , P.‐J. Lin , H.‐H. Lu , C.‐W. Kuo , C. M. Chang , A. D. Hall , E. I. Vivas , J.‐W. Shui , P. Chen , T. A. Hacker , F. E. Rey , T. J. Kamp , P. C. H. Hsieh , Circulation 2019, 139, 647. 30586712 10.1161/CIRCULATIONAHA.118.035235

[45] S. Bohl , D. J. Medway , J. Schulz‐Menger , J. E. Schneider , S. Neubauer , C. A. Lygate , Am. J. Physiol.: Heart Circ. Physiol. 2009, 297, H2054. 19820193 10.1152/ajpheart.00836.2009 PMC2793132

[46] T. Ago , J. Kuroda , J. Pain , C. Fu , H. Li , J. Sadoshima , Circ. Res. 2010, 106, 1253. 20185797 10.1161/CIRCRESAHA.109.213116 PMC2855780

[47] L. Schoefer , R. Mohan , A. Schwiertz , A. Braune , M. Blaut , Appl. Environ. Microbiol. 2003, 69, 5849. 14532034 10.1128/AEM.69.10.5849-5854.2003 PMC201214

[48] M. C. Runtsch , S. Angiari , A. Hooftman , R. Wadhwa , Y. Zhang , Y. Zheng , J. S. Spina , M. C. Ruzek , M. A. Argiriadi , A. F. McGettrick , R. S. Mendez , A. Zotta , C. G. Peace , A. Walsh , R. Chirillo , E. Hams , P. G. Fallon , R. Jayaraman , K. Dua , A. C. Brown , R. Y. Kim , J. C. Horvat , P. M. Hansbro , C. Wang , L. A. J. O'Neill , Cell Metab. 2022, 34, 487. 35235776 10.1016/j.cmet.2022.02.002

[49] J. Wang , H. Du , W. Xie , J. Bi , H. Zhang , X. Liu , Y. Wang , S. Zhang , A. Lei , C. He , H. Yuan , J. Zhang , Y. Li , P. Xu , S. Liu , Y. Zhou , J. Shen , J. Wu , Y. Cai , C. Yang , Z. Li , Y. Liang , Y. Zhao , J. Zhang , M. Song , Circ. Res. 2024, 135, 1161. 39465245 10.1161/CIRCRESAHA.124.325212

[50] Z. Wu , Y. Shi , Y. Cui , X. Xing , L. Zhang , D. Liu , Y. Zhang , J. Dong , L. Jin , M. Pang , R. Xiao , Z. Zhu , J. Xiong , X. Tong , Y. Zhang , S. Wang , F. Tang , B. Zhang , Protein Cell 2023, 14, 350. 37155312 10.1093/procel/pwac010 PMC10166170

[51] W. Peng , Y. Zhang , M. Zheng , H. Cheng , W. Zhu , C.‐M. Cao , R.‐P. Xiao , Circ. Res. 2010, 106, 102. 19910575 10.1161/CIRCRESAHA.109.210914 PMC2815328

[52] M. I. Love , W. Huber , S. Anders , Genome Biol. 2014, 15, 550. 25516281 10.1186/s13059-014-0550-8 PMC4302049

[53] B. Gou , X. Chu , Y. Xiao , P. Liu , H. Zhang , Z. Gao , M. Song , Front. Cardiovasc. Med. 2022, 9, 900978. 35615560 10.3389/fcvm.2022.900978 PMC9124831

[54] D. Damian , M. Gorfine , Nat. Genet. 2004, 36, 663. 15226741 10.1038/ng0704-663a

[55] A. Subramanian , P. Tamayo , V. K. Mootha , S. Mukherjee , B. L. Ebert , M. A. Gillette , A. Paulovich , S. L. Pomeroy , T. R. Golub , E. S. Lander , J. P. Mesirov , Proc. Natl. Acad. Sci. U. S. A. 2005, 102, 15545. 16199517 10.1073/pnas.0506580102 PMC1239896

[56] M. Ackers‐Johnson , P. Y. Li , A. P. Holmes , S.‐M. O'Brien , D. Pavlovic , R. S. Foo , Circ. Res. 2016, 119, 909. 27502479 10.1161/CIRCRESAHA.116.309202 PMC5965670

[57] Q. Chu , F. Liu , Y. He , X. Jiang , Y. Cai , Z. Wu , K. Yan , L. Geng , Y. Zhang , H. Feng , K. Zhou , S.i Wang , W. Zhang , G.‐H. Liu , S. Ma , J. Qu , M. Song , Protein Cell 2022, 13, 676. 35038130 10.1007/s13238-021-00898-9 PMC9233732

[58] C. A. Alvarez‐Breckenridge , J. Yu , R. Price , J. Wojton , J. Pradarelli , H. Mao , M. Wei , Y. Wang , S. He , J. Hardcastle , S. A. Fernandez , B. Kaur , S. E. Lawler , E. Vivier , O. Mandelboim , A. Moretta , M. A. Caligiuri , E. A. Chiocca , Nat. Med. 2012, 18, 1827. 23178246 10.1038/nm.3013 PMC3668784

