# Supplementary material for: Flavonifractor Plautii or Its Metabolite Desaminotyrosine as Prophylactic Agents for Alleviating Myocardial Ischemia/Reperfusion Injury
Source: Adv Sci (Weinh). 2025 Mar 16;12(21):2417827. doi: 10.1002/advs.202417827 (PMC12140293; doi:10.1002/advs.202417827)
Supplement: Supplementary file 1 — Supporting Information [file ADVS-12-2417827-s001.docx]

**SUPPLEMENTAL FIGURES AND FIGURE LEGENDS**


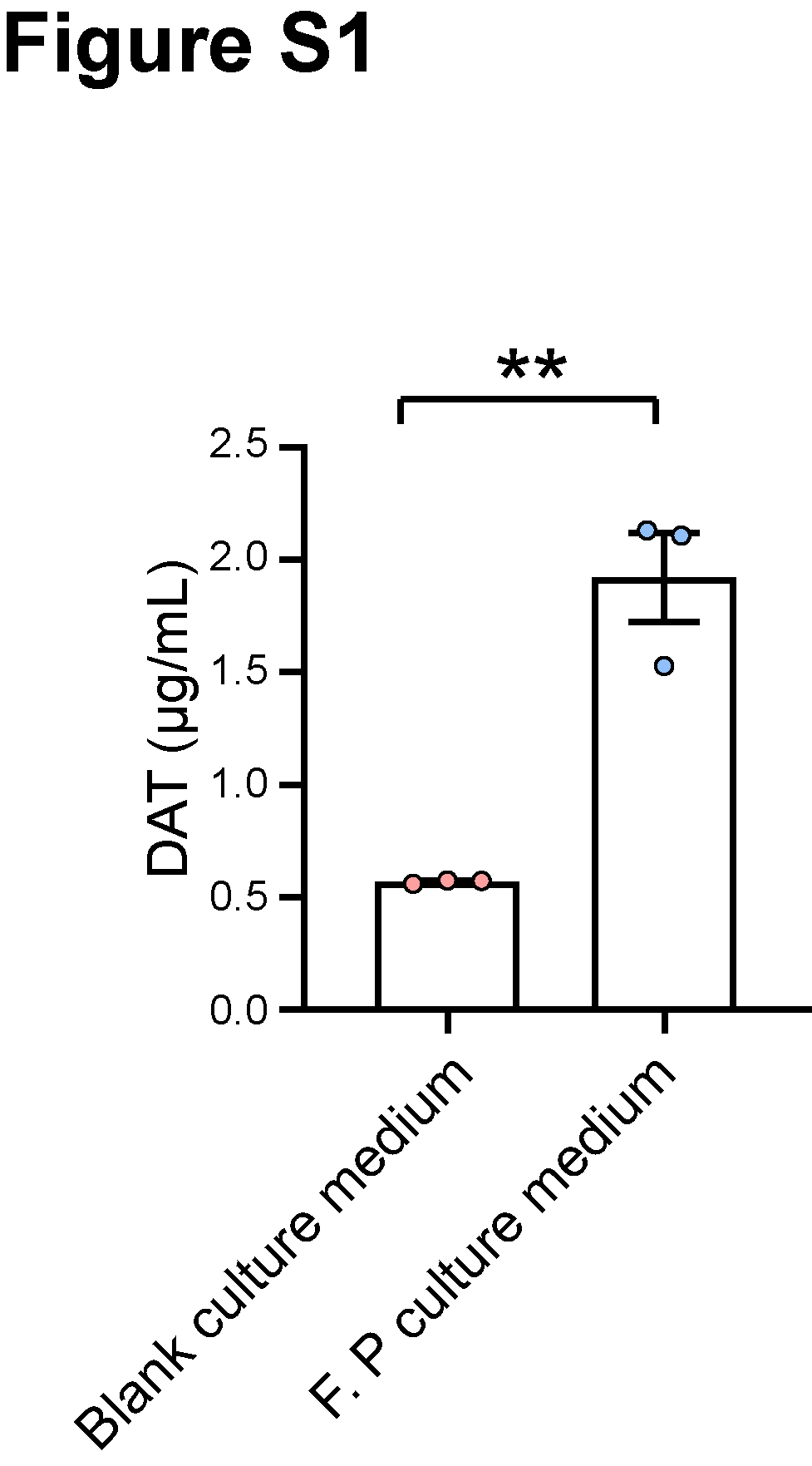


**Figure S1. Measurement of DAT levels by HPLC-MS.**

*In vitro* production of DAT by *F. plautii*. DAT levels were measured in the culture medium of *F. plautii* compared to blank culture medium using HPLC-MS. n = 3 independent experiments. Data are presented as mean ± SEM. ***p*<0.01; (Student′s *t*-test).


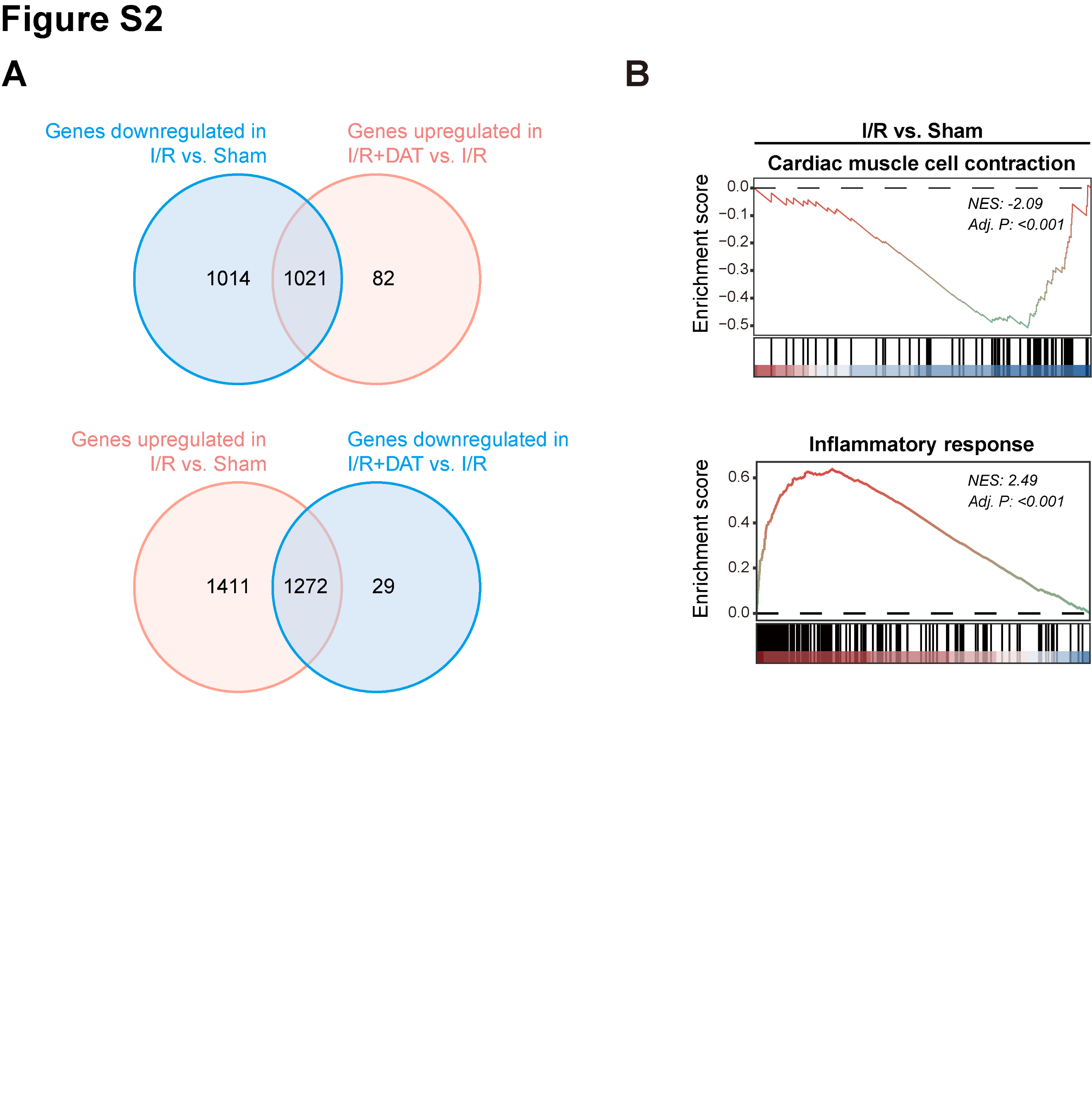


**Figure S2. Transcriptional analysis of DAT’s protective effects against myocardial I/R injury.**

(A) Venn diagram illustrating the overlap between the significantly downregulated genes in I/R versus Sham groups and the significantly upregulated genes in I/R+DAT versus I/R groups (upper panel). Venn diagram illustrating the overlap between the significantly upregulated genes in I/R versus Sham groups and the significantly downregulated genes in I/R+DAT versus I/R groups (lower panel). (B) GSEA plots showing the enrichment of gene sets related to cardiac muscle cell contraction (upper panel) and inflammatory response (lower panel) in I/R versus Sham groups.


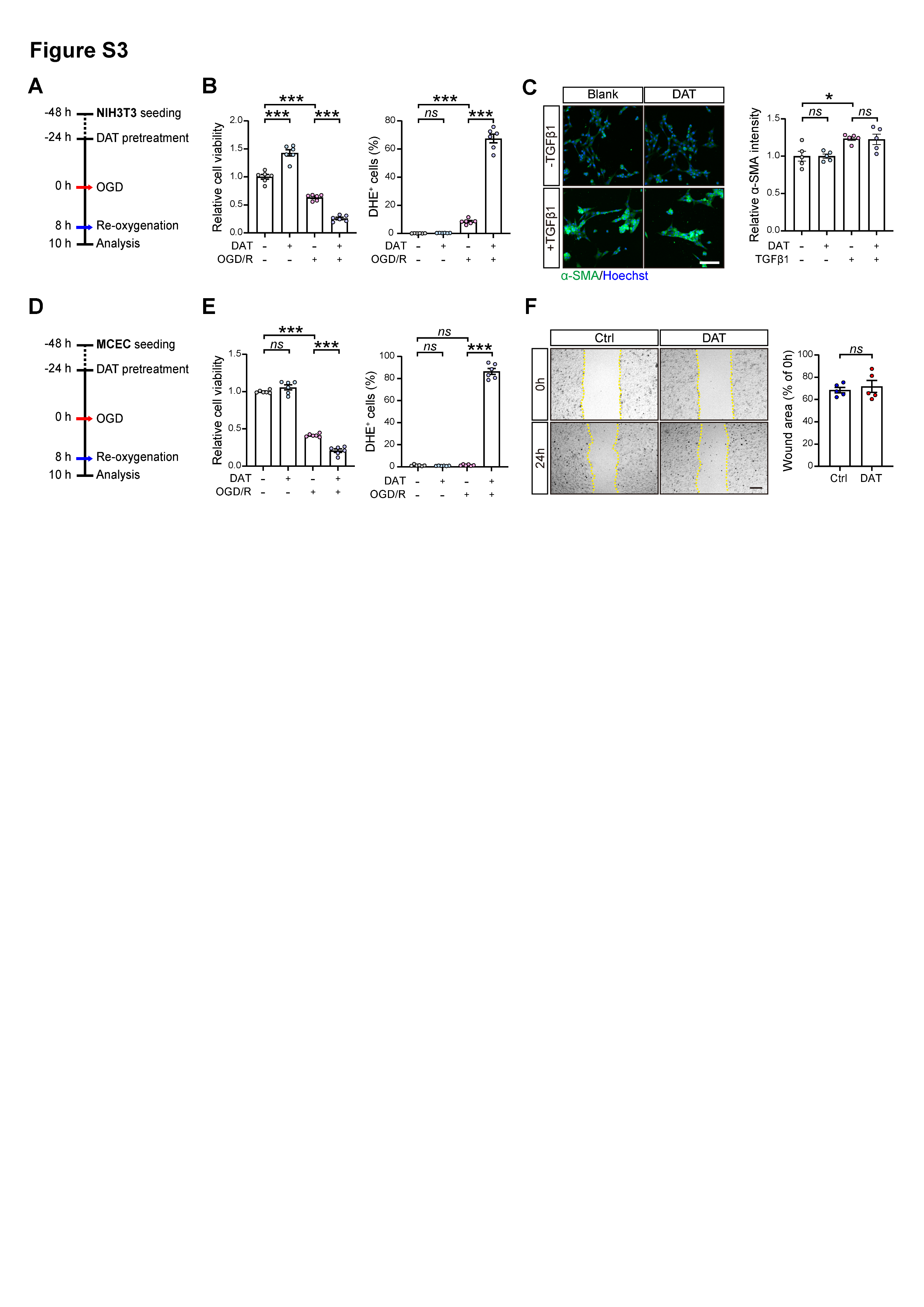


**Figure S3. DAT did not enhance survival or primary functions in NIH3T3 fibroblasts or MCECs.**

(A) Schematic diagram of NIH3T3 fibroblasts subjected to oxygen glucose deprivation/re-oxygenation (OGD/R) that simulated myocardial I/R injury in the presence or absence of DAT pretreatment at 100 μM. (B) Cell viability measured by Alamar blue staining and ROS levels evaluated by DHE staining in NIH3T3 fibroblasts in the presence or absence of DAT treatment at baseline and under OGD/R conditions. *n* = 6 for each group. (C) Immunofluorescence staining of α-SMA in NIH3T3 fibroblasts in the presence or absence of DAT treatment at baseline and upon TGFβ1 stimulation. Scale bar, 100 μm. α-SMA (green) and Hoechst (blue). Quantitative data on fluorescence intensity of α-SMA are shown to the right. *n* = 5 for each group. (D) Schematic diagram of mouse cardiac endothelial cells (MCECs) subjected to OGD/R in the presence or absence of DAT pretreatment at 100 μM. (E) Cell viability measured by Alamar blue staining and ROS levels evaluated by DHE staining in MCECs in the presence or absence of DAT treatment at baseline and under OGD/R conditions. *n* = 6 for each group. (F) Wound healing assay of MCECs in the presence or absence of DAT treatment. Scale bar, 200 μm. The migration rate was determined by measuring the wound area from the initial time to 24 hours after scratch. *n* = 5 for each group. Data are presented as mean ± SEM. Groups were compared using two-way ANOVA (B, C, and E) or Student′s *t*-test (F). *ns*, not significant; **p*<0.05; ****p*<0.001.


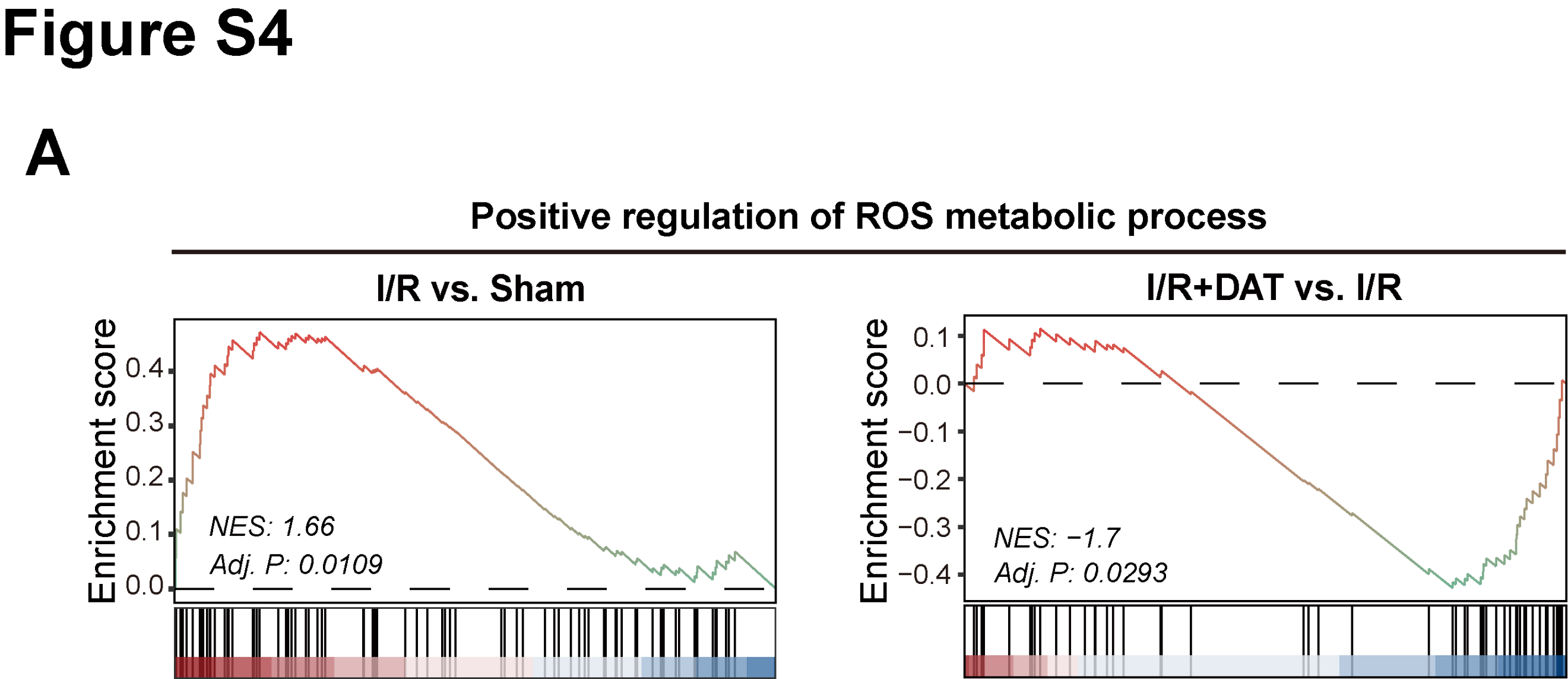


**Figure S4. GSEA showing the regulation on positive regulation of ROS metabolic process in mouse hearts.**

GSEA plots showing the enrichment of gene sets of positive regulation of ROS metabolic process in I/R versus Sham groups (left panel) and in I/R+DAT versus I/R groups (right panel).


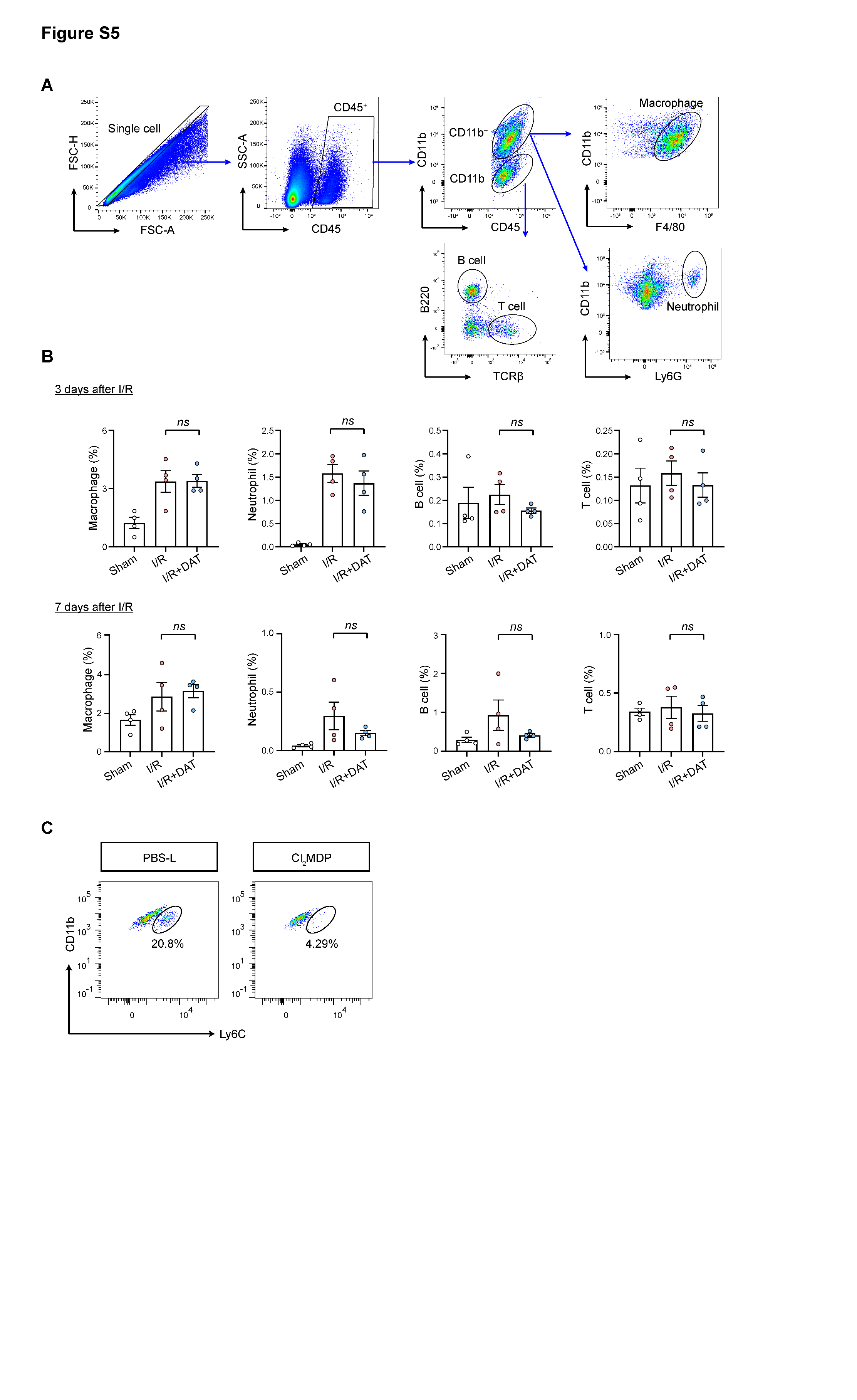


**Figure S5. DAT treatment did not alter the number of immune cell types tested in mouse hearts after I/R.**

(A) Gating strategy identifying several types of immune cells including macrophages, neutrophils, B cells, and T cells in mouse hearts at three days and one week after I/R. (B) Quantification of the percentages of macrophages, neutrophils, B cell and T cell in heart tissue at three days and one week after I/R in all non-myocytes. *n* =4 for each group. Data are presented as mean ± SEM. *ns*, not significant (one-way ANOVA). (C) Representative dot plots showing the depletion of circulating monocytes by clodronate liposomes (Cl_2_MDP) in comparison with those treated with PBS liposomes (PBS-L).


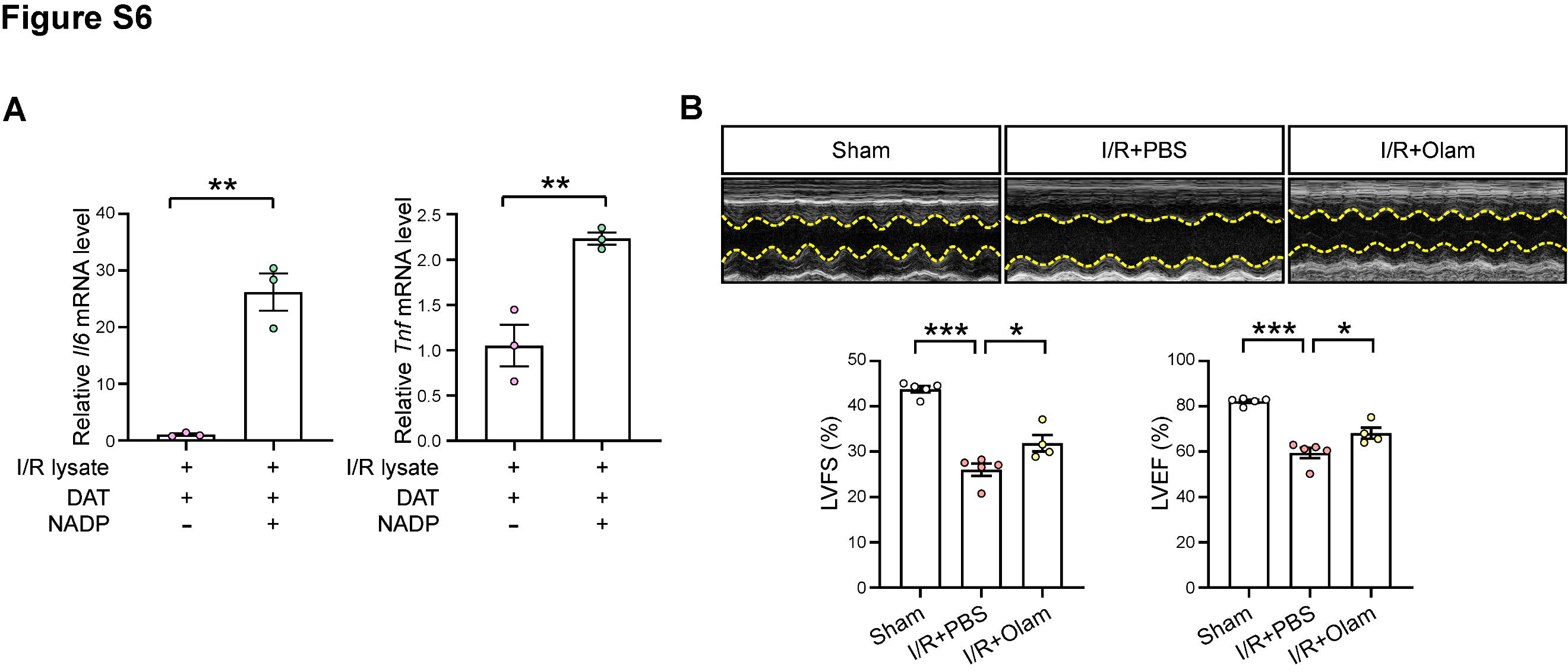


**Figure S6. DAT exerted anti-inflammatory effects on BMDMs in a redox-dependent manner and olamkicept alone attenuated cardiac dysfunction in mouse hearts after I/R.**

(A) qRT‐PCR analysis showing the mRNA levels of *Il6* and *Tnf* in BMDMs stimulated with I/R lysate and pretreated with DAT at 100 μM in the presence or absence of oxidized NADP^+^ (NADP). *n =* 3 for each group. (B) Representative echocardiographic images from Sham, PBS-injected I/R (I/R+PBS), and olamkicept-injected I/R (I/R+olamkicept) groups at one week after the surgery (upper panel). Quantitative data of left ventricular fractional shortening (LVFS) and ejection fraction (LVEF) are shown as mean ± SEM. *n =* 5 for Sham and I/R+PBS groups; *n =* 4 for I/R+olamkicept group. Groups were compared using Student’s *t*-test (A) and one-way ANOVA with post hoc Dunnett's test (B). **p*<0.05; ***p*<0.01; ****p*<0.001.
